# TCF-1 limits the formation of Tc17 cells via repression of the MAF–RORγt axis

**DOI:** 10.1084/jem.20181778

**Published:** 2019-05-29

**Authors:** Lisa A. Mielke, Yang Liao, Ella Bridie Clemens, Matthew A. Firth, Brigette Duckworth, Qiutong Huang, Francisca F. Almeida, Michael Chopin, Hui-Fern Koay, Carolyn A. Bell, Soroor Hediyeh-Zadeh, Simone L. Park, Dinesh Raghu, Jarny Choi, Tracy L. Putoczki, Philip D. Hodgkin, Ashley E. Franks, Laura K. Mackay, Dale I. Godfrey, Melissa J. Davis, Hai-Hui Xue, Vanessa L. Bryant, Katherine Kedzierska, Wei Shi, Gabrielle T. Belz

**Affiliations:** 1 Walter and Eliza Hall Institute of Medical Research, Parkville, Australia; 2 Department of Medical Biology, University of Melbourne, Parkville, Australia; 3 Olivia Newton-John Cancer Research Institute and La Trobe University School of Cancer Medicine, Heidelberg, Australia; 4 Department of Microbiology and Immunology, University of Melbourne, Peter Doherty Institute for Infection and Immunity, Melbourne, Australia; 5 Australian Research Council Centre of Excellence in Advanced Molecular Imaging, University of Melbourne, Melbourne, Australia; 6 Department of Physiology, Anatomy and Microbiology, La Trobe University, Bundoora, Australia; 7 Department of Anatomy and Neuroscience, University of Melbourne, Parkville, Australia; 8 Centre for Future Landscapes, La Trobe University, Bundoora, Australia; 9 Department of Microbiology and Immunology, Carver College of Medicine, University of Iowa, Iowa City, IA; 10 Department of Clinical Immunology & Allergy, The Royal Melbourne Hospital, Parkville, Australia; 11 Department of Biochemistry and Molecular Biology, Faculty of Medicine, Dentistry and Health Sciences, University of Melbourne, Melbourne, Australia; 12 Department of Computing and Information Systems, University of Melbourne, Parkville, Australia

## Abstract

Interleukin (IL)-17–producing CD8^+^ T (Tc17) cells have emerged as key players in host-microbiota interactions, infection, and cancer. The factors that drive their development, in contrast to interferon (IFN)-γ–producing effector CD8^+^ T cells, are not clear. Here we demonstrate that the transcription factor TCF-1 (*Tcf7*) regulates CD8^+^ T cell fate decisions in double-positive (DP) thymocytes through the sequential suppression of MAF and RORγt, in parallel with TCF-1–driven modulation of chromatin state. Ablation of TCF-1 resulted in enhanced Tc17 cell development and exposed a gene set signature to drive tissue repair and lipid metabolism, which was distinct from other CD8^+^ T cell subsets. IL-17–producing CD8^+^ T cells isolated from healthy humans were also distinct from CD8^+^IL-17^−^ T cells and enriched in pathways driven by *MAF* and *RORγt*. Overall, our study reveals how TCF-1 exerts central control of T cell differentiation in the thymus by normally repressing Tc17 differentiation and promoting an effector fate outcome.

## Abstract (precis)

Mielke et al. show that TCF-1 limits IL-17–producing CD8^+^ T (Tc17) cell development from double-positive thymocytes through the sequential suppression of MAF and RORγt, while cementing conventional CD8^+^ T cell fate.

## Introduction

CD8^+^ T cell differentiation traditionally results in the emergence of two subsets: effector cells that produce IFN-γ and memory cells that mediate immune protection against pathogen infections. More recently, it has emerged that other specialized CD8^+^ T cell populations develop in immune responses and are critical to orchestrate complete immune protection. These subsets include tissue-resident memory T cells, follicular cytotoxic T cells, and two CD8^+^ T cell subsets that produce IL-17, namely, mucosal-associated invariant T (MAIT) cells and IL-17–producing CD8^+^ T (Tc17) cells.

Tc17 cells are present in multiple disease settings such as gastrointestinal cancers (Zhuang et al., 2012; Wu et al., 2014) and graft versus host disease (Gartlan et al., 2015). They have been shown to protect against exposure to lethal fungal infection and to respond to microbial colonization in the skin (Naik et al., 2015; Linehan et al., 2018; Harrison et al., 2019) and influenza virus infection (Hamada et al., 2009). Despite these cardinal immunomodulatory roles, the transcriptional circuit that controls Tc17 cell differentiation is not well understood. The acquisition of the ability of a CD8^+^ T cell to produce IL-17 appears to occur when the T-box factors, T-box transcription factor (T-BET) and EOMES (Intlekofer et al., 2008), or T-BET and Blimp (Xin et al., 2016), are down-regulated. RAR-related orphan receptor γ (RORγt) also promotes IL-17 production in CD8^+^ T cells by binding the IL-17 conserved noncoding sequence-2 enhancer region (Ysebrant de Lendonck et al., 2013). However, forced expression of RORγt only results in a small increase in Tc17 cells (Ysebrant de Lendonck et al., 2013), suggesting that while RORγt is necessary, it is not sufficient for IL-17 expression, and other, as yet undefined factors must be required to cooperate with RORγt to drive specification of the Tc17 program.

T cell factor 1 (TCF-1) is a transcription factor previously shown to be critical for differentiation of early thymic progenitor cells (Germar et al., 2011; Weber et al., 2011). More recently it has been shown that TCF-1 also plays a role in thymic T cell development to establish CD4^+^ and CD8^+^ T cell fate (Steinke et al., 2014; Xing et al., 2016). TCF-1 is critical for CD8^+^ memory T cell responses during infection (Jeannet et al., 2010; Zhou et al., 2010; Xing et al., 2016) and more broadly directs the divergence of CD4^+^ T cell lineages into T helper type 2 cells (Yu et al., 2009) and follicular cytotoxic and T follicular helper subsets (Choi et al., 2015; Wu et al., 2015; Xu et al., 2015; Leong et al., 2016), but suppresses the differentiation of T helper type 17 (Th17) cells and promotes their stem cell–like properties (Ma et al., 2011; Yu et al., 2011; Zhang et al., 2018; Karmaus et al., 2019). In situations of chronic stimulation such as cancer or chronic infection, TCF-1 is critical for sustaining CD8^+^ T cell responses (Nanjappa et al., 2012, 2017; Im et al., 2016; Utzschneider et al., 2016; Wu et al., 2016; Kurtulus et al., 2019). Despite the significant impact of TCF-1 at various stages of T cell development, whether it plays a role in the generation of Tc17 cells has not yet been established.

In this study, we demonstrate that the loss of TCF-1 results in the emergence of conventional IL-17–producing CD8^+^ T cells, which we have defined as Tc17 cells. Ablation of TCF-1 during T cell development revealed that divergence to a Tc17 cell subset was constitutive when TCF-1–mediated repression was removed in double-positive (DP) thymocytes. We identify MAF BZIP transcription factor (MAF) and RORγt as critical for the formation of Tc17 cells and as direct targets of TCF-1. Loss of TCF-1 was associated with global changes in the chromatin architecture in CD8^+^ T cells, leading to increased accessibility of *Rorc* and Tc17 effector genes *IL-17a* and *IL-17f* in Tc17 cells. In contrast, we observed reduced accessibility of *Tbx21*, *Eomes*, and *Irf4* loci in Tc17 cells, genes normally required for the induction of effector and central memory CD8^+^ T cells. Finally, we demonstrate that IL-17–producing CD8^+^ T cells are present in healthy humans and express *MAF* and *RORγt*. Collectively, our study reveals the regulatory role of TCF-1 as instrumental in suppressing the MAF–RORγt axis in developing T cells to limit Tc17 formation.

## Results

### TCF-1 restricts Tc17 cell development

**Figure 1.**
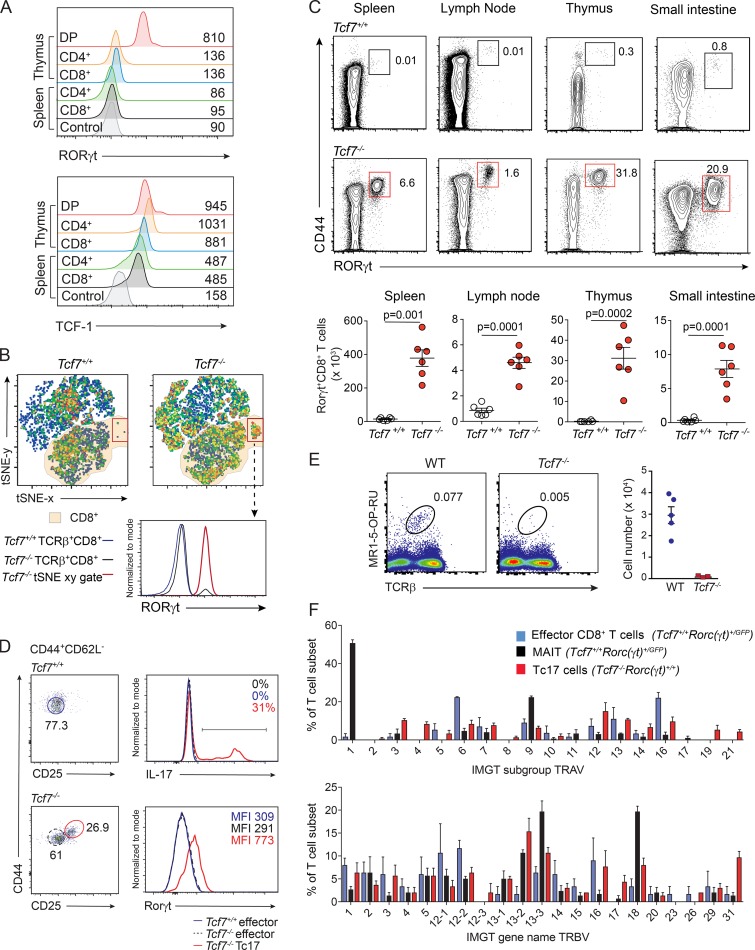
**Loss of TCF-1 results in the emergence of IL-17–producing CD8^+^ T (Tc17) cells. (A)** Expression of RORγt (upper panel) and TCF-1 (lower panel) in various T cell populations from thymus and spleen of WT mice. Populations include DP (TCRβ^−^CD4^+^CD8^+^) thymocytes and CD4^+^ (TCRβ^+^CD4^+^CD8^−^) or CD8^+^ (TCRβ^+^CD4^−^CD8^+^) T cells isolated from spleen or thymus. Control for RORγt expression represents fluorescence minus one stain of CD8^+^ T cells from WT mice and for TCF-1 expression shows spleen CD8^+^ T cells from *Tcf7^−/−^* mice. Data show representative plots of one of two independent experiments (*n* = 4 mice/experiment). **(B)** tSNE analysis of TCRβ^+^ cells analyzed by flow cytometry for CD4, CD8, CD62L, CD44, and RORγt from spleen of *Tcf7^+/+^* and *Tcf7^−/−^* mice. Dot plots are displayed as pseudocolor plots denoting areas of high and low population density. Orange shading indicates CD8^+^ T cells. Histogram shows RORγt expression. Representative of three independent experiments (*n* = 6 mice). **(C)** Contour plots gated on TCRβ^+^CD8^+^CD4^−^ T cells show the frequency (upper panels) and number (lower panel) of RORγt^+^CD8^+^ T cells in various tissues in *Tcf7^+/+^* and *Tcf7^−/−^* mice. Data show representative plots (upper panels) and the mean ± SEM of individuals pooled from three independent experiments (lower panels; *n* = 6, P values calculated using Student’s *t* test). **(D)** IL-17 production and RORγt^+^ expression by *Tcf7^−/−^* Tc17 cells (red, TCRβ^+^CD8^+^CD4^−^CD44^+^CD62L^−^CD25^+^) and *Tcf7^+/+^* or *Tcf7^−/−^* effector cells (blue and black, TCRβ^+^CD8^+^CD4^−^CD44^+^CD62L^−^CD25^−^) FACS-purified from the spleen and lymph nodes of *Tcf7^+/+^* and *Tcf7^−/−^* mice, followed by stimulation with PMA and ionomycin for 4 h in vitro. Histograms are representative of one of two independent experiments (*n* = 3 mice/genotype/experiment). **(E)** Flow-cytometric analyses of MAIT cells in spleen of WT and *Tcf7^−/−^* mice. Data show representative staining from an individual mouse for each genotype and data from one of two similar experiments (*n* = 3–5 mice/genotype). **(F)** Single-cell PCR analysis of TCR Vα (upper panel) and TCR Vβ (lower panel) usage of effector CD8^+^ T cells (TCRβ^+^CD4^−^CD8^+^CD44^+^CD62L^−^*Rorc(*g*t)^+/+^*) and MAIT cells from *Tcf7^+/+^Rorc(*g*t)^+/GFP^* mice (TCRβ^+^CD4^−^CD8^+^CD44^+^CD62L^−^*Rorc(*g*t)^+/GFP^*) and Tc17 cells from *Tcf7^−/−^* mice (TCRβ^+^CD4^−^CD8^+^CD44^+^CD62L^−^CD25^+^). Data show the mean expression ± SEM of 50 individual cells analyzed for TCR Vα and TCR Vβ usage from each of three mice.

Regulation of RORγt expression is dramatically down-regulated as DP thymocytes transition to single-positive (SP) CD4^+^ or CD8^+^ T cells (Fig. 1 A, upper panel; and Fig. S1 A). In contrast, TCF-1 expression is maintained throughout positive selection in the thymus and is not down-regulated until SP naive T cells migrate to the periphery (Fig. 1 A, lower panel). We wanted to determine whether sustained TCF-1 was critical for the dynamic regulation of RORγt expression that is essential for T cell maturation (He et al., 2000). To investigate this, we assessed splenic TCRβ^+^ T cells from 6-wk-old *Tcf7^+/+^* and *Tcf7^−/−^* mice by unbiased clustering t-Distribution Stochastic Neighbor Embedding (tSNE) multiparameter analysis of CD4, CD8, CD44, CD62L, and RORγt staining. While the frequency of a number of populations was altered in the absence of TCF-1, one of the most striking differences was associated with a significant enrichment of RORγt^+^CD8^+^ T cells (Fig. 1 B). These cells were expanded in the thymus, secondary lymphoid tissues, and nonlymphoid tissues such as the small intestine, up to 25-fold across all tissues examined (Fig. 1 C). By contrast, in naive WT mice, very few of these cells were normally detected. RORγt^+^CD8^+^ T cells developing in the absence of TCF-1 had an activated phenotype marked by high CD44 and low CD62L expression, but in addition, they also expressed CD25 (Fig. 1 D and Fig. S1 B), a marker that is not highly expressed in WT mice at steady state. In the absence of TCF-1, these TCRβ^+^CD4^−^CD8^+^CD44^+^CD62L^−^CD25^+^ T cells readily expressed RORγt (Fig. 1 D) and produced IL-17 in response to stimulation ex vivo with PMA and ionomycin (Fig. 1 D). Further characterization did not show any aberrant production of inflammatory cytokines such as IFN-γ, IL-6, IL-22, TNF-α, or IL-2 (Fig. S1 C).

Given that MAIT cells share some features similar to RORγt^+^IL-17^+^CD8^+^ T cells that develop in the absence of TCF-1, including their expression of CD8α, RORγt and IL-17 production (Rahimpour et al., 2015), we examined TCRβ^+^CD4^−^CD8^+^CD44^+^CD62L^+^RORγt^+^ T cells from the spleen of WT and *Tcf7^−/−^* mice for the transcription factor promyelocytic leukemia zinc finger protein (PLZF), a factor essential for MAIT cell development (Koay et al., 2016). In contrast to cells from *Tcf7^+/+^* mice, cells from *Tcf7^−/−^* mice did not express PLZF (Fig. S1 D). Furthermore, in contrast to the RORγt^+^CD8^+^ T cells found in *Tcf7^−/−^* mice, we found that MAIT cells required TCF-1 for their development (Fig. 1 E). Thus, TCRβ^+^CD4^−^CD8^+^CD44^+^CD62L^+^RORγt^+^ T cells found in the spleen of WT mice are MAIT cells and are distinct from those found in *Tcf7^−/−^* mice. This suggests that the RORγt^+^CD8^+^ T cells present in TCF-1-deficient mice are Tc17 cells consistent with recent work showing that Tc17 cells are not present at steady-state in wild-type mice bred in specific pathogen-free facilities (Linehan et al., 2018). Furthermore, Tc17 cells from *Tcf7^−/−^* mice did not show a highly restricted semiinvariant TCR-α chain as featured by MAIT cells (TRAV1–TRAJ33, also designated Vα19-J33; Fig. 1 F). Instead, *Tcf7^−/−^* Tc17 cells expressed a very diverse range of both Vα and Vβ chains (Fig. 1 F). Thus, TCRβ^+^CD4^−^CD8^+^CD44^+^CD62L^+^RORγt^+^ T cells in mice at steady state are predominantly composed of MAIT cells, while TCF-1 deficiency results in the emergence of a distinct subset of Tc17 cells that exhibit a diverse TCR repertoire. To determine if the Tc17 cells we observed were induced by commensal microbes, we treated pregnant mothers before the birth of pups and continued treatment of pups with a cocktail of antibiotics to eliminate bacterial stimulation. This revealed that similar to untreated *Tcf7^−/−^* mice, Tc17 cells develop despite the ablation of bacterial communities (Fig. S1 E). To further examine if the Tc17 cells that develop in the absence of TCF-1 could emerge in response to microbe-derived peptides recognized by the nonclassical MHC class Ib molecules, H2-M3 as seen in previous work (Linehan et al., 2018), we probed Tc17 cells for their ability to bind H2-M3 tetrameric complexes loaded with the *Staphylococcus epidermidis*–derived N-formyl methionine peptide, MIIINA. CD8^+^ T cells isolated from the spleen and ears of naive *Tcf7^+/+^* and *Tcf7^−/−^* mice together with WT mice that had been infected with *S. epidermidis* for 7 d were examined (Fig. S1 F). Although f-MIIINA:H2-M3 tetramer–positive cells were enriched in the spleen and ears of *S. epidermidis*–associated mice, *S. epidermidis*–specific cells were not expanded in these tissues in *Tcf7^−/−^* mice (Fig. S1 F). Collectively, these data demonstrate that the Tc17 cells that emerge in the absence of TCF-1 are not driven by the normal intestinal microbiota, and TCF-1 plays a critical role in suppressing development of these Tc17 cells.

### TCF-1 suppresses the transcriptional profile associated with IL-17–producing cells

**Figure 2.**
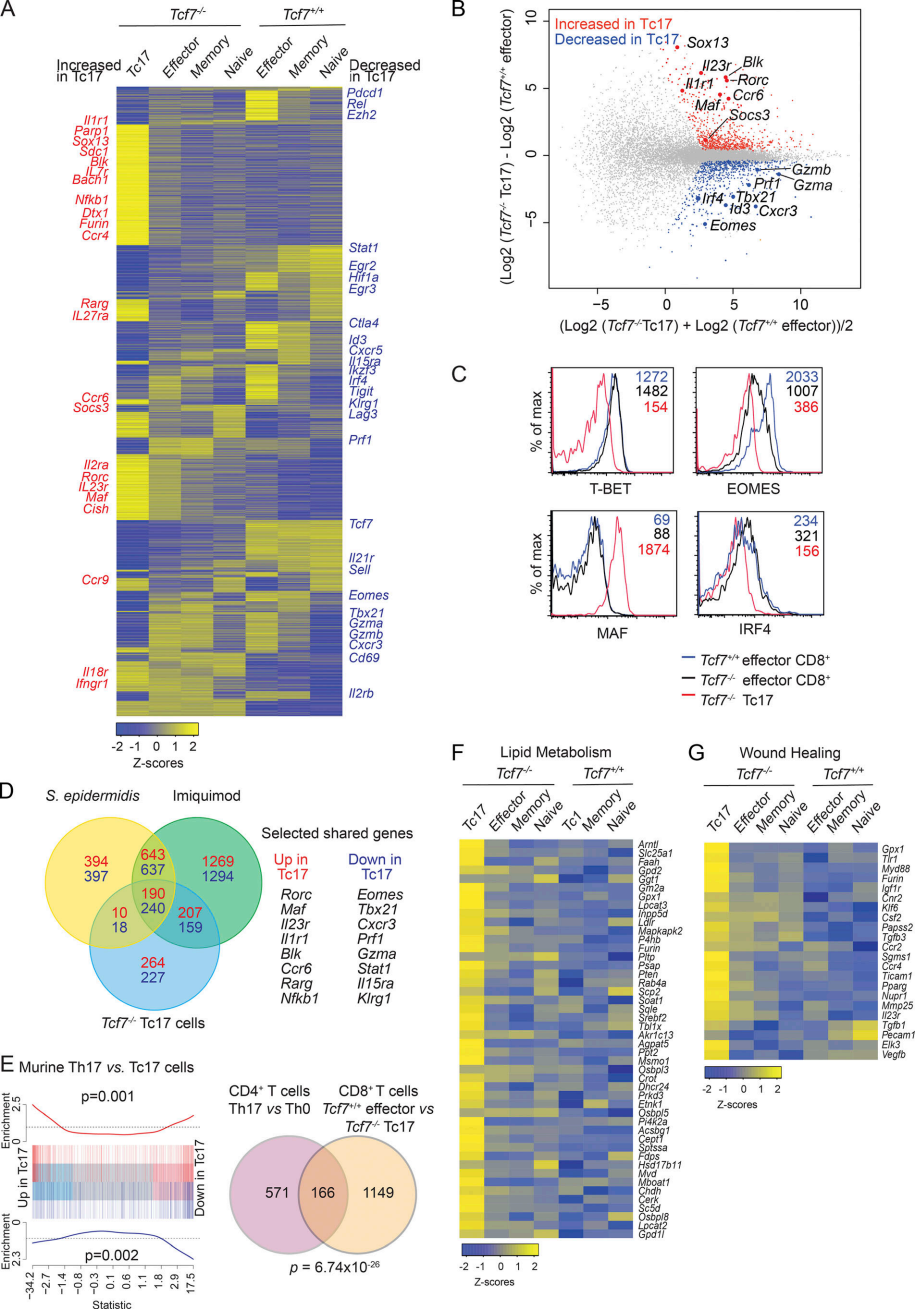
**TCF-1–deficient Tc17 cells are transcriptionally distinct from other CD8^+^ T cell subsets. (A)** Heat map showing DE genes by Tc17 cells (TCRβ^+^CD4^−^CD8^+^CD44^+^CD62L^−^CD25^+^) compared with naive (TCRβ^+^CD4^−^CD8^+^CD44^−^CD62L^+^CD25^−^), effector (TCRβ^+^CD4^−^CD8^+^CD44^+^CD62L^−^CD25^−^), and memory (TCRβ^+^CD4^−^CD8^+^CD44^+^CD62L^+^CD25^−^) CD8^+^ T cell populations from *Tcf7^+/+^* and *Tcf7^−/−^* mice. Relative expression (Z-scores) of genes are shown; rows are scaled to have a mean of 0 and an SD of 1. For each T cell population, two independent biological replicates were analyzed. Data show the mean expression values for each group. **(B)** Mean-difference plot comparing *Tcf7^−/−^* Tc17 cells with *Tcf7^+/+^* effector cells showing up-regulated genes in red and down-regulated genes in blue. Data show log2-fold change compared with mean expression. **(C)** Transcriptional profile of Tc17 cells. Intracellular analyses of the expression of T-BET, EOMES, MAF, and IRF4 in WT effector cells, *Tcf7^−/−^* effector cells, and Tc17 cells. Effector CD8^+^ T cells were identified as TCRβ^+^CD4^−^CD8^+^CD44^+^CD62L^−^RORγt^−^ (effector WT, blue; *Tcf7^−/−^*, black), while Tc17 cells were TCRβ^+^CD4^−^CD8^+^CD44^+^CD62L^−^RORγt^+^ (*Tcf7^−/−^,* red). Data show the MFI of expression for each CD8^+^ T cell population and show representative profiles from one of two similar experiments (*n* = 4 mice/genotype). **(D)** Venn diagram analysis of shared genes among Tc17 cell types. Comparison of *Tcf7^−/−^* Tc17 versus WT effector CD8^+^ T cells DE genes with DE genes in imiquimod-induced skin Tc17 cells and DE genes in topical *S. epidermidis*–induced Tc17 cells. Number of genes up-regulated in Tc17 cells compared with effector cells for each comparison is shown in red, and the number of down-regulated genes is shown in blue. **(E)** Comparison between RNA-seq analyses of murine T helper type 0 versus Th17 cells from a published dataset (GSE40918, eight libraries; Ciofani et al., 2012) with *Tcf7^+/+^* effector versus *Tcf7^−/−^* Tc17 cells. **(F and G)** Heat map analysis showing up-regulated genes in Tc17 cells enriched for lipid metabolism (F)or wound healing (G) in Tc17 cells compared with naive, effector, and memory CD8^+^ T cell populations from *Tcf7^+/+^* and *Tcf7^−/−^* mice. Relative expression (Z-scores) of genes are shown, color coded according to the legend. Rows are scaled to have a mean of 0 and an SD of 1.

To identify the molecular mechanisms underlying the emergence of Tc17 cells, we compared their gene expression profile to effector and other CD8^+^ T cell subsets. We took advantage of our finding that Tc17 cells express CD25 and performed RNA-sequencing (RNA-seq) on FACS sorted naive (TCRβ^+^CD4^−^CD8^+^CD44^−^CD62L^+^CD25^−^), effector (TCRβ^+^CD4^−^CD8^+^CD44^+^CD62L^−^CD25^−^), and memory CD8^+^ T cells (TCRβ^+^CD4^−^CD8^+^CD44^+^CD62L^+^CD25^−^) isolated from *Tcf7^+/+^* and *Tcf7^−/−^* mice and Tc17 cells (TCRβ^+^CD4^−^CD8^+^CD44^+^CD62L^−^CD25^+^) from *Tcf7^−/−^* mice (Fig. 2). Multidimensional scaling analysis (Fig. S2 A) and heat map (Fig. 2 A) analysis revealed that Tc17 cells had a distinct transcriptional profile compared with naive, effector, and memory CD8^+^ T cells from both *Tcf7^+/+^* mice and *Tcf7^−/−^* mice (Fig. 2 A). Given that Tc17 cells had an activated phenotype (CD44^+^CD62L^−^) but are not present in WT mice at steady state (Fig. 1 and Linehan et al., 2018), we compared them directly to the expression profile of WT effector cells. We identified 671 genes up-regulated in Tc17 cells compared with WT effector cells and 644 down-regulated genes (Fig. 2 B). Genes were called differentially expressed (DE) if they achieved an FDR of ≤0.05. Among the up-regulated genes was the transcription factor *Rorc*, a critical factor required for all IL-17–producing cell types (Santori et al., 2015; Fig. 2, A and B). Also included in this list of up-regulated genes were genes that have previously been shown to a play role in regulating IL-17 production in CD4^+^ T cells or γδ T cells such as *Maf, Sox13*, and *Blk* (Bauquet et al., 2009; Laird et al., 2010; Gray et al., 2013; Tanaka et al., 2014), the expression of which in Tc17 has not previously been described (Fig. 2, A and B). In addition, a number of cytokine and chemokine receptors such as *Il7r, Il27ra, Il23r, Il1r1, Ccr4, Ccr6*, and *Ccr9* were up-regulated by Tc17 cells compared with naive, effector, and memory CD8^+^ T cells (Fig. 2 A). Further investigation of transcription factors known to control effector cell differentiation revealed that *Tbx21, Id3, Irf4,* and *Eomes* were down-regulated in Tc17 cells compared with WT effector cells (Fig. 2, A and B). These observations were corroborated by flow cytometry for protein expression of T-BET, EOMES, MAF, and IRF4 (Fig. 2 C). Our analyses of *Tbx21/Eomes* double knockout mice did not show increased constitutive Tc17 cells at steady state (data not shown). This is consistent with previous reports showing that T-BET and EOMES suppress the differentiation of IL-17–producing CD8^+^ T cells following lymphocytic choriomeningitis infection but exhibited normal T cell composition at steady state (Intlekofer et al., 2005, 2008). This analysis identifies TCF-1 as a critical transcriptional repressor of Tc17 cell differentiation and normally preserves the expression of genes associated with conventional effector CD8^+^ T cell differentiation.

### *Tcf7*-deficient Tc17 cells and pathogen-induced Tc17 cells exhibit a conserved transcriptional profile that is distinct to Th17 cells

Our data and others show that Tc17 cells are not present in WT mice housed in SPF facilities at steady state (Linehan et al., 2018). Although we show that *Tcf7^−/−^* Tc17 cells do not recognize the f-MIIINA:H2-M3, we investigated whether transcriptionally these cells were similar to or different from the Tc17 cells shown to develop in the skin following imiquimod treatment or in association with the commensal bacteria *S. epidermidis* (Linehan et al., 2018). Gene set enrichment analysis between our RNA-seq data and the published dataset (Linehan et al., 2018) showed a high correlation between genes DE in *Tcf7^−/−^* Tc17 cells and both imiquimod (Fig. S2 B) and *S. epidermidis*–induced (Fig. S2 C) Tc17 cells (P < 0.0001 in all comparisons). Venn diagram analysis comparing all three Tc17 cell types showed that 190 genes highly expressed in *Tcf7^−/−^* Tc17 cells were shared among all three Tc17 cell types, including the transcription factors *Rorc* and *Maf* (Fig. 2 D). In addition, 240 shared genes were down-regulated in all three Tc17 cell types including *Tbx21* and *Eomes*, transcription factors previously shown to suppresses Tc17 development following lymphocytic choriomeningitis virus infection (Intlekofer et al., 2008). To understand how similar or distinct *Tcf7^−/−^* Tc17 cells are from Th17 cells, we compared RNA-seq data of murine T helper type 0 and Th17 cells from a published dataset (Ciofani et al., 2012) to identify the Th17 specific gene set. This was then compared with the DE gene list from our own Tc17 analyses (Fig. 2 E). This revealed that while Th17 and Tc17 cells share a common gene set as would be expected of IL-17–producing cells (166 genes), a large proportion (87%) of genes were distinct from those expressed by Th17 cells (1,149 genes). Thus, Tc17 cells express a unique transcriptional program that establishes their regulation as distinct from Th17 cells.

### Tc17 cells exhibit enhanced capacity for lipid metabolism and tissue repair

To understand how Tc17 cells are distinct from other T cell subsets, we used gene network analyses to uncover novel features of these cells. Metascape analyses (http://metascape.org; Tripathi et al., 2015) of genes down-regulated in Tc17 cells revealed a dampening of pathways associated with T cell activation, immune effector function, negative regulation of the immune system, and cell adhesion (Fig. S2 D). Interrogation of up-regulated genes revealed gene clusters associated with lipid metabolism, inflammation, and immune cell development (Fig. S2 E). Lipid metabolism plays an integral role in the survival, proliferation, and differentiation of pathogenic Th17 cells (Berod et al., 2014) but has not previously been described for Tc17 cells. Comparison of the Tc17 profile with other CD8^+^ T cell subsets revealed that genes involved in phospholipid and glycerophospholipid metabolism were specifically enriched in Tc17 cells (Fig. 2 F). This included target genes of the sterol response element binding proteins (*Srebp*) and master regulators of fatty acid and cholesterol synthesis, including *Gpd2, Ldlr, Soat1, Sqle, Srebf2*, and *Mvd* (Walls et al., 2016). Intermediates of this pathway have recently been shown to act as agonists for RORγt (Hu et al., 2015; Santori et al., 2015). Further gene ontology analyses revealed an enrichment in genes involved in wound healing (Log10 (P) = −6.6), particularly genes involved in chemotaxis (*Ccr2, Csf2, Tgfb1, Tgfb3,* and *Furin*), angiogenesis (*Vegfb* and *Csf2*), and tissue remodeling (*Mmp25*), was increased in Tc17 cells compared with all other CD8^+^ T cell subsets (Fig. 2 G). This is consistent with a recent study in which tissue repair pathways have been shown to be increased in Tc17 cells (Linehan et al., 2018; Harrison et al., 2019).

### TCF-1 regulates chromatin architecture to suppress Tc17 cell potential

**Figure 3.**
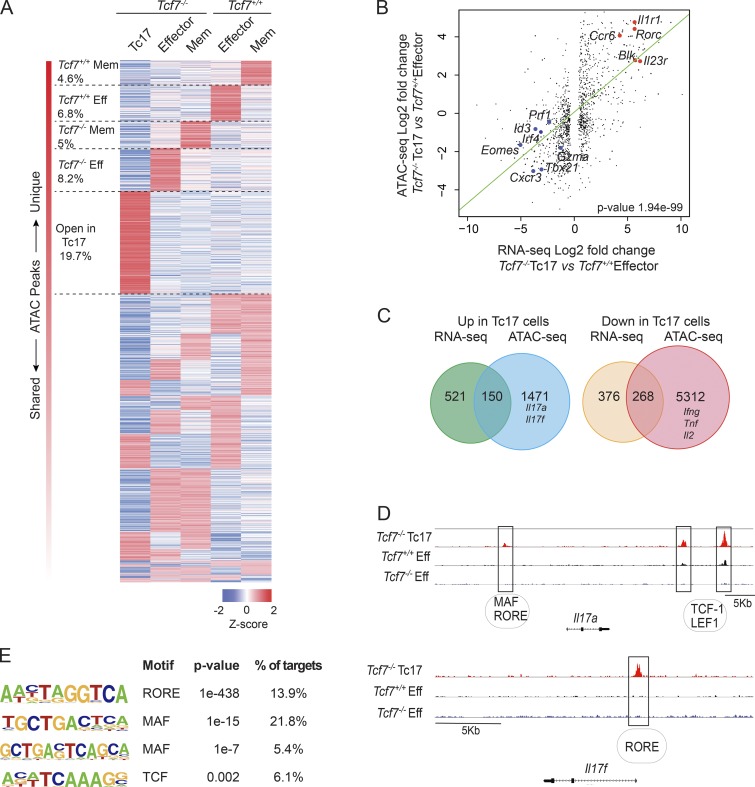
**TCF-1 maintains the chromatin landscape of CD8^+^ T cells to suppress Tc17 potential. (A)** Heat map of regions of chromatin accessibility across the genomes of *Tcf7^−/−^* Tc17 cells (TCRβ^+^CD4^−^CD8^+^CD44^+^CD62L^−^CD25^+^), effector (TCRβ^+^CD4^−^CD8^+^CD44^+^CD62L^−^CD25^−^) and memory (TCRβ^+^CD4^−^CD8^+^CD44^+^CD62L^+^CD25^−^) CD8^+^ T cells from *Tcf7^+/+^* and *Tcf7^−/−^* mice. Average relative peak abundance (Z-score) calculated from two independent biological replicates from log2-CPM value of peak regions. **(B)** Correlation analysis of DE genes from RNA-seq analysis of *Tcf7^+/+^* effector CD8^+^ T cells and *Tcf7^−/−^* Tc17 cells (x axis) with gene accessibility (y axis). The green line is the regression line with a slope of 0.48. P value of the linear regression analysis is shown in the figure. **(C)** Overlap of DE genes from RNA-seq analysis and genes associated with differentially accessible chromatin regions in *Tcf7^−/−^* Tc17 cells and *Tcf7^+/+^* effector CD8^+^ T cells. **(D)** Genome browser of normalized ATAC-seq reads across the *Il17a* and *Il17f* loci from *Tcf7^−/−^* Tc17 and effector CD8^+^ T cells from *Tcf7^+/+^* and *Tcf7^−/−^* mice. Predicted transcription factor binding sites are listed below as assessed by using the evolutionary rate covariance browser. **(E)** De novo motif analysis using HOMER in regions of chromatin with increased accessibility in Tc17 cells. Eff, effector CD8^+^ T cells; Mem, memory CD8^+^ T cells.

In addition to TCF-1 directly binding DNA, this factor has recently been shown to have intrinsic histone deacetylase activity, capable of coordinating the opening of chromatin in early T cell development, and to repress CD4^+^ T cell fate in CD8^+^ T cells (Xing et al., 2016; Johnson et al., 2018). This provides a second process by which TCF-1 is able to regulate gene expression. We therefore hypothesized that TCF-1 regulates chromatin accessibility as a mechanism to suppress Tc17 cell potential. To this end, we performed Assay for Transposase-Accessible Chromatin (ATAC) sequencing (ATAC-seq) on *Tcf7^−/−^* Tc17 cells, effector and memory CD8^+^ T cells purified by flow-cytometric sorting from *Tcf7^+/+^* and *Tcf7^−/−^* mice. Our unsupervised clustering analysis revealed that 19.7% of regulatory elements were more accessible and unique to Tc17 cells compared with only 8.2% of *Tcf7^−/−^* Tc1, 5% of *Tcf7^−/−^* memory, 6.8% of *Tcf7^+/+^* effector, and 4.6% of *Tcf7^+/+^* memory T cells (Fig. 3 A), thus suggesting that Tc17 constitute a distinct CD8^+^ T cell subset. These accessible regions were assigned to a gene if they were located within 20 kilobase pairs upstream of the transcription start site or 5 kbp downstream of the 3′ end of gene loci. To determine the strength of the relationship between TCF-1–regulated genes identified by our RNA-seq, we correlated gene expression with ATAC-seq peak scores for each DE gene (Fig. 3 B). A significant positive correlation (regression slope, 0.48; P value, 1.94 ×10^−99^) emerged between accessible chromatin and DE genes in *Tcf7^−/−^* Tc17 cells and *Tcf7^+/+^* effector cells (Fig. 3 B). Analysis of the chromatin accessibility of loci encoding key transcription factors associated with T cell differentiation showed that *Rorc* was more accessible in Tc17 cells (Fig. 3 B and Fig. S3 A), while transcription factors with decreased gene expression also have decreased chromatin accessibility (e.g., *Eomes, Tbx21, Id3,* and *Irf4*; Fig. 3 B and Fig. S3 A). Thus, TCF-1 appears to be a major molecular switch controlling chromatin accessibility of key genes associated with the divergence between effector cells and Tc17 cells.

### Tc17 cells are primed for IL-17 production

Our ATAC-seq analysis revealed that 1,726 regions of chromatin (1,621 genes) were more accessible in *Tcf7^−/−^* Tc17 cells compared with *Tcf7^+/+^* effector CD8^+^ T cells. Most of these genes (1,471) were not associated with genes actively expressed in Tc17 cells at steady state (Fig. 3 C). These included accessible chromatin regions associated with Tc17 effector genes such as *Il17a* and *Il17f* (Fig. 3 C). We found that many more genes (5,580) were less accessible in Tc17 cells and open in effector cells. Of these, 5,312 were not associated with actively expressed genes in effector cells, including *Ifng, Tnf*, and *Il2* (Fig. 3 C and Fig. S3 B). A close examination of the *Il17a* locus revealed three regions with increased accessibility in Tc17 cells compared with effector CD8^+^ T cells (Fig. 3 D). Using the ECR Browser (http://ecrbrowser.dcode.org), we found the stretch of DNA upstream of the *Il17a* loci contained MAF binding motifs and ROR response elements (RORE), predicted to bind RORγt (Fig. 3 D, upper panel). Two regions were also identified in the distal region of *Il17a* that were more accessible in Tc17 cells compared with effector cells (Fig. 3 D) containing numerous TCF-1 and LEF-1 binding motifs (Fig. 3 D). The promoter region of *Il17f* also displays increased accessibility in *Tcf7^−/−^* Tc17 cells (Fig. 3 D, lower panel), containing RORE binding motifs. These results show that the *Il17* loci are poised for gene expression in Tc17 cells developing in the absence of TCF-1, but remained closed in effector cells from both WT and TCF-1–deficient mice. Finally, a global analysis of transcription factor binding motifs revealed that RORE and MAF binding motifs were among the most enriched in accessible chromatin regions specific to Tc17 cells (Fig. 3 E and Table S1). Given that RORγt and MAF were among the most highly expressed transcription factors in Tc17 cells (Fig. 2 A), we hypothesized that lack of TCF-1 releases suppression of RORγt and MAF to orchestrate Tc17 differentiation.

### Early deletion of TCF-1 drives constitutive formation of Tc17 cells

TCF-1 is essential for establishing T cell development in both the thymus and periphery (Yu et al., 2009, 2011; Ma et al., 2011; Xu et al., 2015; Leong et al., 2016; Xing et al., 2016; Johnson et al., 2018). Nevertheless, the mechanisms underpinning Tc17 cell development in the absence of TCF-1 remain unclear. To determine where loss of TCF-1 influenced the emergence of Tc17 cells, we generated two different mouse strains, allowing us to address the temporal consequences of TCF-1 loss at different stages of T cell development. To understand the impact of TCF-1 loss at the early stages, *Tcf7^fl/fl^* mice were crossed with *Cd4Cre^+/T^* mice (Sawada et al., 1994; *Tcf7^fl/fl^Cd4Cre^+/T^*), resulting in the loss of TCF-1 at the DP stage in the thymus (Fig. S4 A). For the second strain, *Tcf7^fl/fl^* were crossed to *dLck^Cre^* mice (*Tcf7^fl/fl^dLck^cre+^*), whereby Cre-recombinase gene expression is driven by the distal promoter of the lymphocyte protein tyrosine kinase (*dLck*; Wang et al., 2001) and induces ablation of TCF-1 after thymic positive selection, resulting in a peripheral CD8^+^ T cell population lacking *Tcf7* (Fig. S4 B).

**Figure 4.**
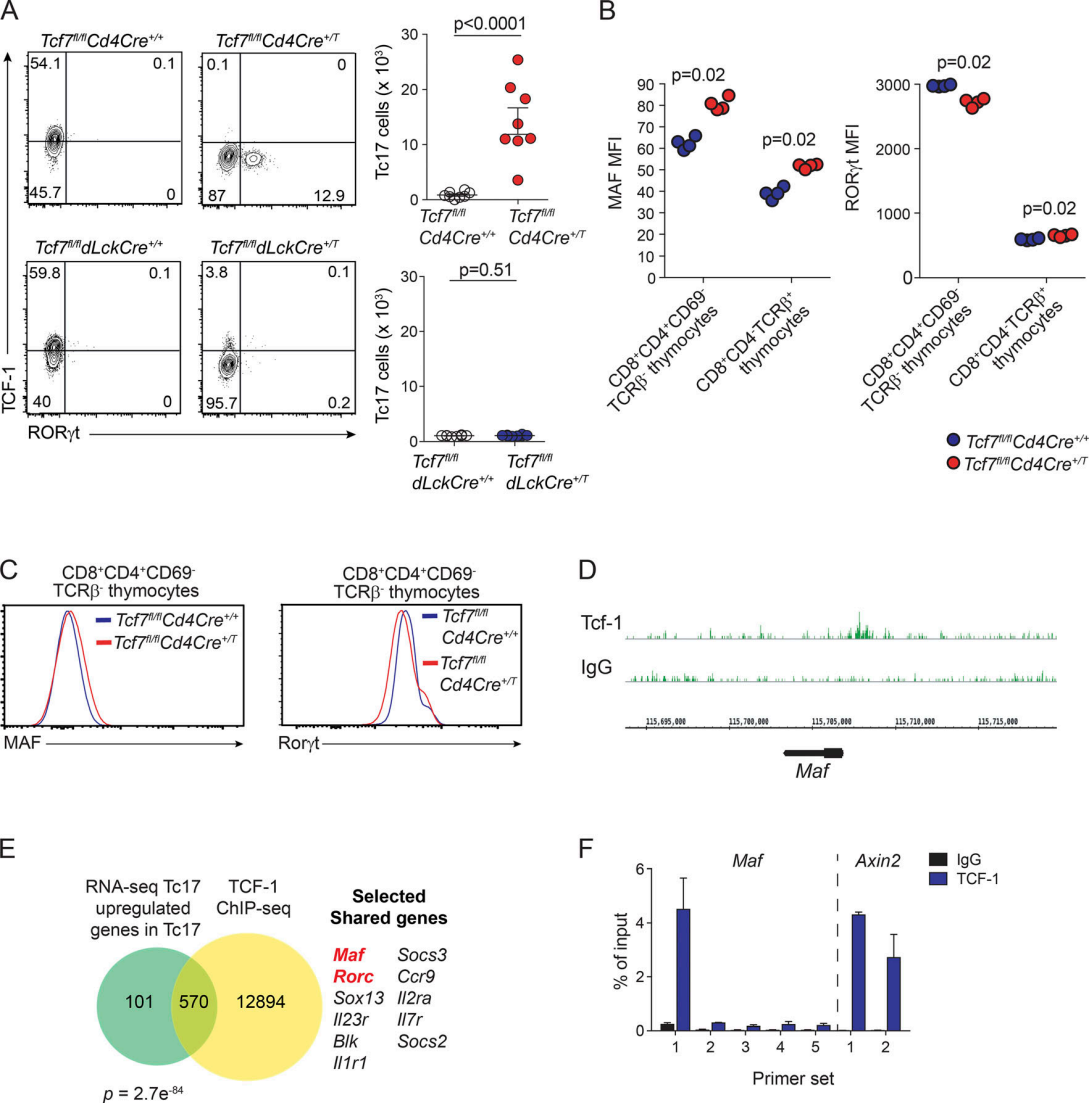
**TCF-1 controls Tc17 generation and MAF expression in developing thymocytes. (A)** Flow-cytometric analyses and enumeration of RORγt and TCF-1 expression in TCRβ^+^CD8^+^CD62L^−^CD44^+^ splenocytes isolated from *Tcf7^fl/fl^CD4Cre^+/+^* and *Tcf7^fl/fl^CD4Cre^+/T^* mice (upper panels) and *Tcf7^fl/fl^dLckCre^+/+^* and *Tcf7^fl/fl^dLckCre^+/T^* mice (lower panels). Data are pooled from two independent experiments and show the mean ± SEM (*n* = 4 or 5 mice/genotype/experiment). Exact P values were calculated using Student’s *t* test. **(B)** Geometric MFI of MAF and RORγt expression in DP thymocytes (CD4^+^CD8^+^TCRβ^−^CD69^−^) and CD8 SP thymocytes (CD4^−^CD8^+^TCRβ^+^). Data are representative of two independent experiments (*n* = 4 mice/group/experiment) and show the mean MFI from one experiment. Exact P values were calculated using the Mann–Whitney *U* test. **(C)** Representative histograms of MAF and RORγt expression in DP thymocytes (CD4^+^CD8^+^TCRβ^−^CD69^−^) from B. **(D)** Genome browser of normalized TCF-1 ChIP-seq reads across the *Maf* locus is shown in green identified by ChIP-seq dataset from Dose et al. (2014) from WT mouse thymocytes. **(E)** Venn diagram analyses of up-regulated genes as determined by RNA-seq of *Tcf7^−/−^* Tc17 cells versus *Tcf7^+/+^* effector cells, compared with TCF-1 target genes identified by ChIP-seq dataset from Dose et al. (2014). FDR of 10^−5^ was applied when calling ChIP-seq peaks. Selected shared TCF-1 target genes DE by Tc17 cells are shown with key transcription factors highlighted in red. **(F)** ChIP analysis of TCF-1 binding in *Maf* loci of mouse thymocytes. TCF-1 binding in the *Axin2* loci is shown as a positive control. Data show the mean ± SD pooled from two independent experiments.

Analysis of *Tcf7^fl/fl^Cd4Cre^+/T^* mice revealed the constitutive presence of Tc17 cells in the periphery (Fig. 4 A, upper panel). These results confirm that TCF-1 suppression of Tc17 cells is intrinsic to the T cells and is not driven by the lymphopenic environment of germline TCF-1–deficient mice. In sharp contrast, late deletion of TCF-1 did not impair CD8^+^ T cell differentiation as virtually no Tc17 cells were detected in *dLck*-deleted mice at steady state (Fig. 4 A, lower panel). Our analysis of Th17 cells is consistent with a previous report of Zhang et al. (2018) showing that TCF-1 also acts to suppress Th17 cells in *Tcf7^−/−^* mice (Fig. S4 C). However, in contrast to Tc17 cells, in Th17 cells, this occurs before expression of the CD4 coreceptor, as we did not observe an increase in Th17 cells in *Tcf7^fl/fl^Cd4Cre^+/T^* mice compared with *Tcf7^fl/fl^Cd4Cre^+/+^* control mice (Fig. S4 D). Collectively, these findings highlight the different role TCF-1 plays in suppressing Th17 versus Tc17 differentiation. Thus, continuous expression of *Tcf7* in the thymus is critical to repress the constitutive emergence of Tc17 cells before thymic positive selection.

### TCF-1 directly suppresses *Maf* expression in developing thymocytes

We show that TCF-1 plays a critical role in suppressing Tc17 cell development following deletion in DP thymocytes. Both MAF and RORγt were highly expressed in Tc17 cells, and MAF and RORγt binding motifs were significantly enriched in accessible chromatin regions of Tc17 cells. Therefore, we determined whether TCF-1 controls expression of RORγt and MAF at the DP stage of T cell development in the thymus by analyses of *Tcf7^fl/fl^Cd4Cre^+/+^* and *Tcf7^fl/fl^Cd4Cre^+/T^* mice. We observed a slight reduction in the number of DP (CD8^+^CD4^+^CD69^−^TCRβ^−^) thymocytes in *Tcf7^fl/fl^Cd4Cre^+/T^* mice but no reduction in SP CD8^+^ T cells (TCRβ^+^CD8^+^CD4^−^; Fig. S4 E). Interestingly, when analyzing these same populations for MAF expression, we observed a significant increase in MAF expression in DP thymocytes of *Tcf7^fl/fl^Cd4Cre^+/T^* mice (Fig. 4 B, left panel; and Fig. 4 C). Increased expression was maintained as they matured to SP CD8^+^ T cells (Fig. 4 B, left panel). Consistent with previous reports (Xing et al., 2016; Johnson et al., 2018), in the absence of TCF-1, RORγt expression was reduced in DP thymocytes compared with control mice (Fig. 4 B, right panel; and Fig. 4 C, right panel), and expression was increased in SP CD8^+^ T cells (Fig. 4 B, right panel). These results show that TCF-1 suppresses MAF expression in developing thymocytes and places MAF upstream of RORγt in development of Tc17 cells in TCF-1–deficient mice.

Previous reports investigating the development of Tc17 cells have not shown whether MAF is required for development of these cells, nor whether TCF-1 could be a direct regulator of MAF expression. Therefore, we wanted to determine if TCF-1 could directly bind the promoter region of MAF as a potential mechanism to suppress its expression. Analysis of TCF-1 chromatin immunoprecipitation (ChIP)-sequencing (ChIP-seq) of thymocytes (Dose et al., 2014) revealed that TCF-1 normally binds the promoter DNA regions of *Maf* (Fig. 4 D). Comparison of genes up-regulated in Tc17 cells and direct TCF-1 target genes in thymocytes revealed that 570 genes were bound by TCF-1, including *Maf* and *Rorc* (Fig. 4 E). Analysis of TCF-1–bound genes that were down-regulated in Tc17 cells identified 512 genes including *Tbx21*, *Irf4*, *Id3, Prf1, Klrg1*, and *Gzma* (Fig. S4 F). We confirmed direct binding of TCF-1 to *Maf* loci at the same site identified in the ChIP-seq analysis by performing additional ChIP coupled with quantitative PCR (ChIP-qPCR) analyses for TCF-1 in mouse thymocytes (Fig. 4 F). Collectively, these results demonstrate that TCF-1 binds directly to the gene loci of *Maf* to limit its expression in developing T cells.

### Tc17 cells are present in healthy humans

**Figure 5.**
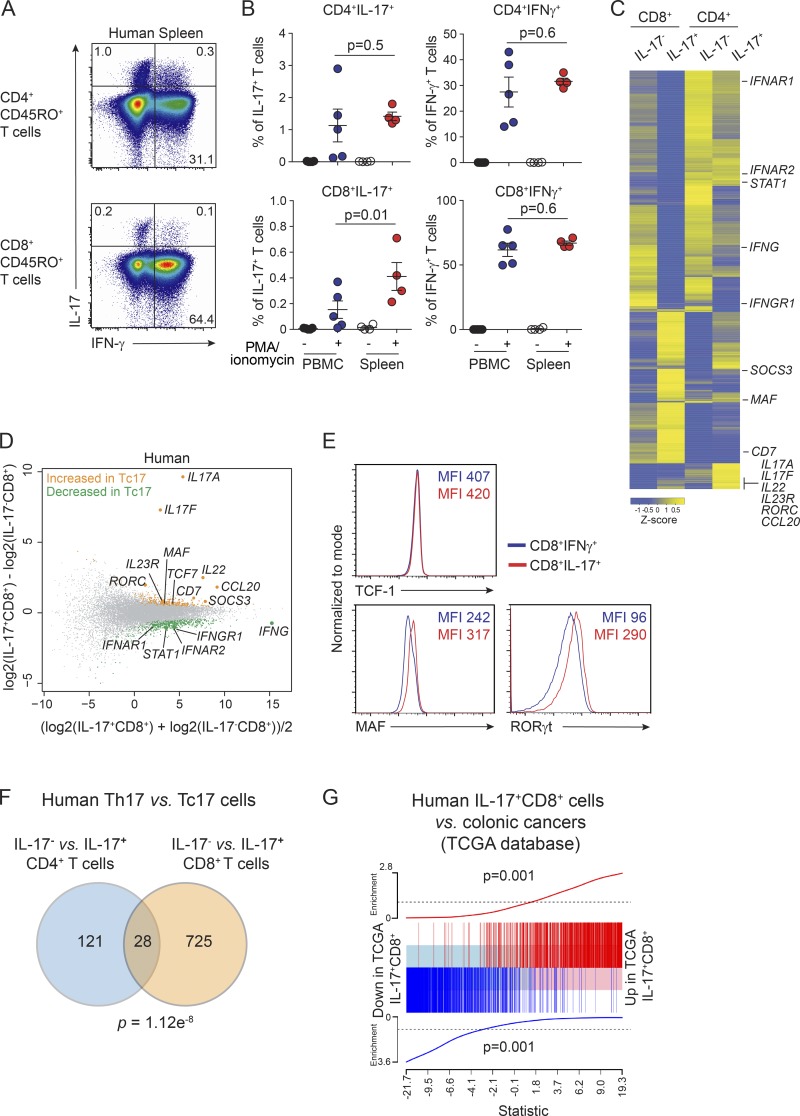
**Tc17 cells occur in healthy humans and express *MAF* and *RORC.* (A)** Representative dot plot analysis from four individual donors of IL-17 and IFN-γ production by T cells from human spleen. Cells are gated on CD45RO^+^CD4^+^ T cells (CD3^+^CD4^+^CD8^−^Vα7.2^−^TCRγδ^−^CD45RO^+^) or CD45RO^+^CD8^+^ T cells (CD3^+^CD4^−^CD8^+^Vα7.2^−^TCRγδ^−^CD45RO^+^). **(B)** Total cell numbers of IL-17 or IFN-γ–producing CD45RO^+^CD4^+^ T cells (upper panel) or CD45RO^+^CD8^+^ T cells (lower panel) from peripheral blood mononuclear cells (*n* = 5 donors) and spleen cells (*n* = 4 donors) from healthy donors determined by intracellular flow cytometry staining following stimulation with PMA and ionomycin for 4 h. Cells are gated as in A. Results are pooled from four independent experiments. Individual responses are shown together with the mean ± SEM; exact P values were calculated using Student’s *t* test. **(C)** Heat map displaying DE genes. Yellow color represents high expression, and blue represents low expression in effector IL-17^−^CD8^+^ versus IL-17^+^CD8^+^ T cells and IL-17^−^CD4^+^ versus IL-17^+^CD4^+^ T cells. Cells were sorted from spleens of three healthy donors for RNA-seq using a human IL-17 cytokine capture kit following restimulation with PMA and ionomycin. IL-17^+^CD8^+^ T cells (CD3^+^CD4^−^CD8^+^Vα7.2^−^TCRγδ^−^IL-17^+^), IL-17^−^CD8^+^ effector T cells (CD3^+^CD4^−^CD8^+^Vα7.2^−^TCRγδ^−^IL-17^−^), IL-17^+^CD4^+^ (CD3^+^CD4^+^CD8^−^Vα7.2^−^TCRγδ^−^IL-17^+^), and IL-17^−^CD4^+^ cells (CD3^+^CD4^+^CD8^−^Vα7.2^−^TCRγδ^−^IL-17^−^). **(D)** Mean difference plot highlighting genes significantly increased in human spleen IL-17^+^CD8^+^ T cells (yellow) versus genes increased in human spleen IL-17^−^CD8^+^ T cells (green). Data show log2-fold change compared with mean expression. **(E)** Protein expression of TCF-1, MAF and RORγt as assessed by flow cytometry from spleen cells of healthy donors. Cells are gated on CD3^+^CD4^−^CD8^+^Vα7.2^−^TCRγδ^−^IFN-γ^+^ (blue) or CD3^+^CD4^−^CD8^+^Vα7.2^−^TCRγδ^−^IL-17^+^ (red). Data show representative histograms from two independent experiments and MFI. **(F)** Comparison of RNA-seq analyses of human IL-17^−^CD4^+^ versus IL-17^+^CD4^+^ with IL-17^−^CD8^+^ versus IL-17^+^CD8^+^ T cells. **(G)** Enrichment of up-regulated (Up signature) and down-regulated (Down signature) genes in human splenic IL-17^+^CD8^+^ cells in the colonic cancers identified from the TCGA database (Up signature P value = 0.001; Down signature P value = 0.001).

Our results and other reports show that Tc17 cells are very rare in mice at steady state (Hamada et al., 2009; Naik et al., 2015), but they appear to occur at higher frequency in mice from the wild, in humans, and under inflammatory conditions (Hamada et al., 2009; Zhuang et al., 2012; Wu et al., 2014; Gartlan et al., 2015; Naik et al., 2015; Amicarella et al., 2017; Linehan et al., 2018). To further investigate Tc17 cells in humans, we analyzed IL-17 and IFN-γ production in T cells from blood and spleen of healthy donors after in vitro stimulation with PMA and ionomycin followed by intracellular staining. MAIT cells and γδ T cells were excluded using antibodies for Vα7.2 and the γδ T cell receptor, respectively (Fig. S5 A). While CD4^+^ T cells (CD3^+^CD4^+^CD8^−^CD45RO^+^Vα7.2^−^TCRγδ^−^) were the main producers of IL-17 (Fig. 5 A, upper panel), a small but consistent number of IL-17^+^CD8^+^ cells were detected (CD3^+^CD4^−^CD8^+^CD45RO^+^Vα7.2^−^TCRγδ^−^IL-17^+^; Fig. 5 A, lower panel). Comparison of IL-17^+^CD8^+^ cells in human blood and spleen demonstrated a slightly higher frequency of these cells in human spleen (Fig. 5 B).

### Human Tc17 cells express *MAF* and *RORC*

Next we analyzed whether human IL-17^+^CD8^+^ T cells have a distinct gene expression profile compared with IL-17^−^CD8^+^ T cells. We used flow cytometry to purify human Tc17 cells from healthy spleen using a human IL-17 cytokine capture kit following a brief (3 h) restimulation with PMA and ionomycin. IL-17^+^CD8^+^ T cells (CD3^+^CD4^−^CD8^+^Vα7.2^−^TCRγδ^−^IL-17^+^), IL-17^−^CD8^+^ T cells (CD3^+^CD4^−^CD8^+^Vα7.2^−^TCRγδ^−^IL-17^−^) along with the analogous CD4^+^ T cell populations, IL-17^+^CD4^+^ T cells (CD3^+^CD4^+^CD8^−^Vα7.2^−^TCRγδ^−^IL-17^+^) and IL-17^−^CD4^+^ T cells (CD3^+^CD4^+^CD8^−^Vα7.2^−^TCRγδ^−^IL-17^−^), were isolated for genomic analyses by RNA sequencing. Our analysis of DE genes (FDR <0.1) revealed 296 genes down-regulated in IL-17^+^CD8^+^ T cells and 457 up-regulated genes compared with IL-17^−^CD8^+^ T cells. Heat map analysis highlighted the unique gene expression profile of IL-17^+^CD8^+^ T cells compared with IL-17^−^CD8^+^ T cells, IL-17^−^CD4^+^ T cells, and IL-17^+^CD4^+^ T cells (Fig. 5 C). *IL17A* and *IL17F* were the two most DE genes when human IL-17^+^CD8^+^ T cells or IL-17^+^CD4^+^ T cells were compared with the respective control IL-17^−^CD8^+^ T cells or IL-17^−^CD4^+^ T cells (Fig. 5 D and Fig. S5 B), indicating that the cytokine capture approach effectively isolated IL-17–producing cells. Importantly, signaling molecules *IL23R* and *SOCS3*, which were significantly up-regulated in murine IL-17^+^CD8^+^ T cells, were also expressed by human IL-17^+^CD8^+^ T cells (Fig. 5 D). In addition, we observed an increase in secreted cytokines *IL22*, the chemokine *CCL20*, and cell surface receptor *CD7* (Fig. 5 D). Analysis of the transcription factors *RORC* and *MAF,* which we identified as highly expressed in mouse Tc17 cells and regulated by TCF-1, showed that they were also expressed by human IL-17^+^CD8^+^ T cells (Fig. 5, D and E). Collectively, these results show that IL-17^+^CD8^+^ T cells, which exist in healthy humans, are Tc17-like and share a similar gene profile to murine Tc17 cells but are distinct from human IL-17^−^CD8^+^ cells, which lack *RORγt* and *MAF* expression.

### Human Tc17 cells are distinctly regulated compared with Th17 cells

As both Tc17 cells and Th17 cells express similar molecular regulators, we investigated whether Tc17 cells exhibited a distinctly separate transcriptional program or were regulated via a highly conserved program between the two T cell subsets. Therefore, we compared our IL-17–producing human CD4^+^ and CD8^+^ T cell subsets analyzed by RNA-seq in this study. We observed a significant overlap (P = 1.12 ×10^−8^) with genes DE between IL-17^−^CD4^+^ and IL-17^+^CD4^+^ cells, and genes DE between IL-17^−^CD8^+^ and IL-17^+^CD8^+^ cells. Despite this, an overwhelming 96% of genes DE were specific to IL-17^+^CD8^+^ cells (Fig. 5 F). Thus, collectively, these analyses strongly support that while CD4^+^ and CD8^+^ IL-17–producing cells share a core set of genes, in humans, Tc17-like cells exist, and these IL-17^+^CD8^+^ cells express a unique transcriptional program that establishes their regulation as distinct from Th17-like IL-17^+^CD4^+^ cells.

IL-17 has been shown to promote progression of colorectal cancer (Grivennikov et al., 2012; Hyun et al., 2012; Wang et al., 2014). Therefore, to determine whether we could identify colorectal cancer samples likely to have infiltrating Tc17 cells, we analyzed 593 patient samples from The Cancer Genome Atlas (TCGA; https://www.cancer.gov/about-nci/organization/ccg/research/structural-genomics/tcga) database. Using Nearest Shrunken Centriod (Tibshirani et al., 2002) and Random Forest (Liaw and Weiner, 2002) classification algorithms, we developed gene expression–based classifiers for the CD8^+^IL-17^+^ Tc17-like cells from our human RNA-seq data. We then used these classifiers to query the TCGA Colon and Rectal Cancer samples to identify those samples with evidence in their transcriptome of a Tc17 expression profile. The samples from TCGA that were predicted by both independent classification methods to resemble Tc17 cells represent the set most likely to contain Tc17 infiltrating cells. To validate the likely Tc17 infiltration in these TCGA samples, we next tested whether these samples are enriched for genes DE in Tc17 splenic cells in our RNA-seq data. We separated putative Tc17 infiltrated samples from other tumor samples, and performed a differential expression analysis between these human tumor sets, then used gene set enrichment analysis to probe the gene expression data for the Tc17 gene expression signature. A significant enrichment of Tc17 genes (1,621 total shared genes between Tc17 cells and colorectal cancers, P = 0.001) was detected amonst these samples (Fig. 5 G). This gene set was distinct from those detected for IL-17^−^CD8^+^, IL-17^+^CD4^+^, and IL-17^−^CD4^+^ T cells, indicating that Tc17 cells exhibit a expression pattern and transcriptional profile separate from other CD8^+^ and CD4^+^ T cell subsets, including the related Th17 cells. Furthermore, this signature is identifiable in a small subset of colonic cancer samples.

### MAF and RORγt drive Tc17 differentiation

**Figure 6.**
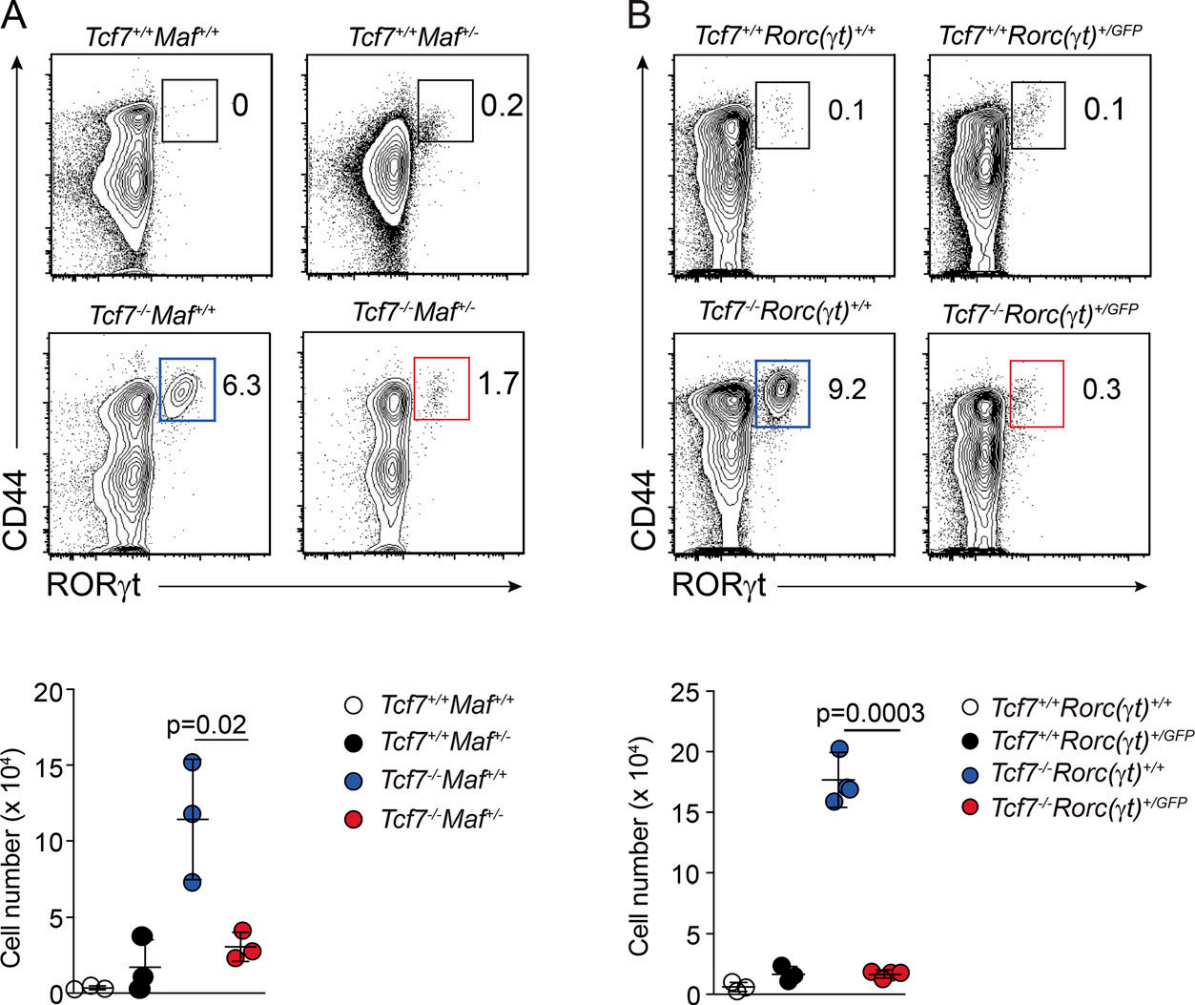
***Maf*and *Rorc* are required for Tc17 differentiation. (A)** Enumeration of Tc17 cells in WT or *Tcf7*^−/−^*c-Maf^+/−^* mice. Contour plots show the frequency of RORγt-expressing cells within live TCRβ^+^CD4^−^CD8^+^ T cells (upper panel). Scatter plots show the total number of TCRβ^+^CD4^−^CD8^+^RORγt^+^ Tc17 cells per spleen for each genotype (lower panel). **(B)** Frequency and total number of Tc17 cells in WT and *Tcf7*^−/−^*Rorc(γt)^+/GFP^* mouse strains. Contour plots show the frequency of RORγt-expressing cells within live TCRβ^+^CD4^−^CD8^+^ T cells (upper panel). Scatter plots show the total number of TCRβ^+^ CD4^−^CD8^+^RORγt^+^ Tc17 cells per spleen for each genotype (lower panel). **(A and B)** Lower panels data show individual mice (mean ± SD) pooled from three independent experiments (*n* = 1 or 2 mice/group/experiment). Exact P values were calculated using Student’s *t* test.

Here we identified increased expression of MAF in TCF-1–deficient DP thymocytes and showed that TCF-1 can bind the promoter region of *Maf*. To investigate the role of *Maf* in Tc17 cell development and to determine whether interaction of TCF-1 with other genes identified in our RNA-seq experiments, namely *Rorc*, also play a role in the development of Tc17 cells, we crossed the *Tcf7^−/−^* mouse strain to the *Maf^+/−^* heterozygous and *Rorc(γt)^gfp^* reporter lines. The mice were analyzed for the constitutive formation of Tc17 cells (Fig. 6, A and B). In MAF-deficient mice, loss of a single copy of *Maf* resulted in a nearly fourfold reduction in the generation of Tc17 cells (Fig. 6 A). Strikingly, the Tc17 population was almost completely ablated when a single copy of *Rorc(γt)* was deleted (Fig. 6 B). Thus, *Maf* and *Rorc(γt)* both play important roles in the development of Tc17 cells at steady state in the absence of TCF-1. Collectively, these data demonstrate that TCF-1 suppresses Tc17 production through the sequential regulation of the transcription factors MAF and RORγt.

## Discussion

The transcriptional network required for the differentiation of Tc17 cells is largely unknown. Deciphering these networks may have important clinical implications to enable manipulation of CD8^+^ T cell differentiation to promote wound healing, resolve infection, and direct T cell responses in cancer (Hamada et al., 2009; Zhuang et al., 2012; Wu et al., 2014; Naik et al., 2015; Nanjappa et al., 2015; Linehan et al., 2018). We show that TCF-1 bound directly to DNA regulatory regions of MAF and RORγt and can limit the expression of MAF and RORγt to suppress the emergence of Tc17 cells. This uncovered a mechanism by which the sequential supression of MAF and RORγt by TCF-1 centrally in the thymus determines the CD8^+^ T cell fate outcome of maturing thymocytes. Indeed, sustained expression of TCF-1 was observed to be necessary for expression of genes important for effector and memory CD8^+^ T cells, while TCF-1 loss enabled divergence of DP thymocytes into Tc17 cells.

TCF-1 plays critical roles in the development of T cells both in the thymus (Okamura et al., 1998; Germar et al., 2011; Weber et al., 2011; Yu et al., 2012) and in peripheral cells (Zhou et al., 2010; Lin et al., 2016). Here we focus on the role of TCF-1 in the thymus, where it is critical to suppress Tc17 cell differentiation, while deletion in mature T cells did not affect Tc17 cell number or frequency. Despite this, how TCF-1 expression shapes the heterogeneity of fate outcomes in the thymus has not been fully elucidated. Our results show that loss of TCF-1 activates the MAF/RORγt circuit during thymic differentiation to guide CD8^+^ T cell heterogeneity.

MAF has been shown to be important in CD4^+^ T cell subsets, particularly in the generation of regulatory T cells (Xu et al., 2018) and follicular helper T cells (Bauquet et al., 2009; Kroenke et al., 2012), and to promote Th17 differentiation (Tanaka et al., 2014). In γδ T cells, MAF antagonizes TCF-1 and LEF-1 to promote the formation and maintenance of RORγt^+^ IL-17–producing γδ T cells (Zuberbuehler et al., 2019). However, the role of MAF in CD8^+^ T cells is not well characterized and to our knowledge has not previously been described in Tc17 cells. We show for the first time that MAF is critical for Tc17 cells, and TCF-1 acts to suppress *Maf* expression in the thymus. Recent evidence shows that TGF-β can drive MAF expression to promote exhaustion of conventional αβ CD8^+^ T cells in melanoma, resulting in defective anti-tumor responses (Giordano et al., 2015). TGF-β has been implicated in Tc17 differentiation in vitro (Hamada et al., 2009; Huber et al., 2013), but it remains to be determined whether TGF-β links the TCF-1–MAF–RORγt axis in Tc17 cells. In T cells, we identified potential TCF-1 binding sites and increased chromatin accessibility in the distal region of *Il17a.* This is consistent with previous reports in CD4^+^ T cells, where TCF-1 has been shown to directly bind *Il17a* regulatory regions and suppress IL-17 production (Yu et al., 2011; Zhang et al., 2018). Our data build on this, showing that TCF-1 also limits expression of the transcription factor RORγt in TCRβ^+^CD8^+^ T cells. Our data further extend this by uncovering a pathway placing MAF upstream of RORγt, normally repressed by TCF-1 to limit the emergence of Tc17 cells.

Transcription factor activity and intrinsic histone deacetylase activity of TCF-1 appear to be essential for coordinating the opening of chromatin early in T cell development, and to establish the dichotomy between CD4^+^ and CD8^+^ T cells (Xing et al., 2016; Johnson et al., 2018). Here, we extend these findings and show that in addition to regulating central T cell lineage choices, TCF-1 suppresses Tc17 differentiation. We found that TCF-1 has a fundamental role in establishing chromatin architecture of CD8^+^ T cells and suppressed the opening of 1,726 chromatin regions in Tc17 cells. We predict some of these will be directly involved in the differentiation of Tc17 cells. TCF-1 was also instrumental in establishing the chromatin landscape of effector cells by cementing three times as many open chromatin regions in effector CD8^+^ T cells. Thus, these results revealed that TCF-1 plays a global role in defining the chromatin landscape of CD8^+^ T cells.

We found that Tc17 cells were significantly enriched for pathways that regulate phospholipid and glycerophospholipid metabolism, in striking contrast to both WT and *Tcf7^−/−^* naive, effector, and memory CD8^+^ T cell subsets. Lipid metabolism is indispensable for generating the main components of cell membranes, provides energy, and is necessary for cell signaling (Howie et al., 2018). These same pathways are exploited by Th17 cells and tumor cells to enhance their metabolic rate and survival (Berod et al., 2014). The gene expression profile of metabolic genes exhibited by Tc17 cells suggests that these cells have requirements distinct from canonical CD8^+^ T cells that fuel subset differentiation. Interestingly, Th17 cells also exhibit a metabolic profile skewed toward phospholipid metabolism (Berod et al., 2014), and these pathways have recently been shown to produce ligands for RORγt (Hu et al., 2015; Santori et al., 2015), further highlighting the potential importance of lipid metabolism for Tc17 cells.

In humans, IL-17 plays a key role in defense against fungal infections (Puel et al., 2011), while several reports have correlated IL-17–producing cells with the development of cancer, including colorectal and gastric cancer (Zhuang et al., 2012; Wu et al., 2014). While this is the case, these studies have generally not clearly distinguished between Tc17, Th17, MAIT, or γδ T cells in humans or assessed their phenotype in detail. We start to delineate a potential role for Tc17 cells in human colorectal cancers by generating a gene expression signature of human Tc17 cells to test in TCGA datasets. We were able to identify a Tc17 signature in a small subset of colorectal cancer samples, indicating that this T cell subset plays a possible role in this disease. We show that Tc17 cells are present in healthy human blood and spleen and exhibit a transcriptional profile that is distinct from IL-17^−^CD8^+^ T cells. The expression profile of human Tc17 cells aligned with our analyses of Tc17 cells in mouse and Tc17 cells that develop in response to imiquimod and commensal bacteria *S. epidermidis* (Naik et al., 2015; Linehan et al., 2018). Thus, the development of Tc17 cells appears to be conserved between man and mouse.

Tc17 cells are emerging as critical players in host-commensal microbiota interactions, infection, and cancer. Despite this, the molecular program that underpins their generation in favor of effector cells has not been previously defined. Together, our work identifies TCF-1 as a core transcriptional switch that in combination with MAF and RORγt defines the Tc17 fate outcome in developing thymocytes. This is mediated through the genome-wide programing of chromatin architecture and suppression of Tc17 identity genes. Thus, these findings are important in identifying the target pathways regulating Tc17 cells that may have important clinical implications for promoting tissue repair, homeostatic preservation of barrier function, and the elimination of intestinal tumors.

## Materials and methods

### Mice

*Tcf7^−/−^* (Verbeek et al., 1995), *Id2^gfp^* (Jackson et al., 2011), *Rorc(γt)^gfp^* (Eberl et al., 2004), *c-maf^−/−^* (Kawauchi et al., 1999), *Tcf7^fl/fl^* (Steinke et al., 2014), *Cd4Cre* (Sawada et al., 1994), and dLckCre (Zhang and Bevan, 2012) have been previously described and were all maintained on a C57BL/6 (Ly5.2) background (originally derived from the Jackson Laboratory). The *Tcf7^−/−^* line was crossed to the *maf^−/+^* and *Rorc(γt)^gfp+/−^* strains to generate the *Tcf7^−/−^maf^+/−^* and *Tcf7^−/−^Rorc(γt)^gfp+/−^* lines, respectively. *Tcf7^−/−^maf^+/−^* mice were maintained as a heterozygous line on the C57BL/6 background as mice lacking both alleles exhibit early postnatal mortality (Kawauchi et al., 1999). Mice were bred and maintained in-house. All mice were used at 6–8 wk old, and procedures were performed in accordance with approvals for the Animal Ethics Committee of the Walter and Eliza Hall Institute of Medical Research.

### Human samples

Peripheral blood mononuclear cells were isolated from five healthy control donors (Volunteer Blood Donor Registry). Healthy human spleens from four donors were obtained from biopsy and cadaveric organ donors (Victorian Cancer Biobank and New South Wales Organ Transplant Registry). This study was performed according to the principles of the Declaration of Helsinki and was approved by local human research ethics committees (Melbourne Health, 2009.162; Walter and Eliza Hall Institute, 10/02).

### Isolation of lymphocytes and flow cytometry

Spleen, thymus, and lymph node cells were isolated by mashing of organs over a 70-µm filter, and cells were resuspended in PBS. Intestinal lymphoid cells were isolated from the intestine by incubation for 45 min at 37°C in Ca^2+^- and Mg^2+^-free HBSS plus 1 mM EDTA, 15 mM Hepes, and 10% FCS, with gentle shaking for removal of intestinal epithelial cells. Supernatants were discarded, and tissues were then incubated with gentle shaking for 45 min in 1 mg/ml (wt/vol) collagenase type III (Worthington), 200 µg/ml DNase I (Roche), and 0.4 U/ml Dispase (Gibco) in RPMI-1640 medium plus 2% (vol/vol) FCS. Preparations were filtered, and mononuclear cells were isolated on a 40–80% Percoll gradient. Lymphocytes were recovered from the interface.

Single-cell suspensions were stained with the following antibodies to mouse cell surface receptors: CD3 (145-2C110), CD4 (GK1.4), TCRβ (H57-597), TCRγδ (GL3), CD8α (53–6.7), CD45.2 (104), CD44 (IM7), and CD62L (MEL-14). For human cell surface receptors, surface staining was performed with antibodies to CD3 (UCHT1), CD4 (RPA-T4), CD8 (RPA-T8; eBioscience), TCRVα7.2 (3C10; BioLegend), γδTCR (11F2; BD Biosciences), CD45RA (HI100), and CD45RO (UCHL1; eBioscience). Intracellular cytokine staining was performed following stimulation for 4 h with PMA (50 ng/ml) and ionomycin (100 ng/ml) in the presence of Brefeldin A (1 µg). Intracellular staining was performed using the Transcription Factor Staining Buffer Set (eBioscience) with antibodies to murine RORγt (AFKJS-9 or Q31-378), TCF-1 (C63D9), T-BET (4B10), EOMES (Dan11Mag), IRF4 (3E4), MAF (Sym0F1), PLZF (Mags.21F7), IL-17 (Ebio17B7), IFN-γ (XMG1.2), IL-2 (JES6-5H4), IL-6 (MP5-20F3), IL-22 (IL22JOP), and TNF-α (MP6-XT22); or antibodies to human IL-17 (eBio64DEC17) and IFN-γ (45.B3). Single-cell suspensions of mouse thymus, spleen, and lymph node were prepared and stained with PE-mouse MR1–5-OP-RU tetramers (Corbett et al., 2014; Koay et al., 2016). *S. epidermidis*–specific CD8^+^ T cells were tracked via the use of a peptide–MHC tetramer, f-MIIINA:H2-M3 (Linehan et al., 2018). Cells were analyzed using a FACS Canto II or Fortessa X20 (BD Biosciences), and FlowJo software was used for analysis. Flow-cytometric sorting was performed with a FACSAria (BD Biosciences).

### Murine T cell isolation

Murine CD8^+^ T cells were enriched from lymph nodes and/or spleens by negative selection of single-cell suspensions incubated with antibodies against Mac-1 (M1/70), Ter-119, GR1 (RB6-8C5), MHC-II (M5/114), and CD4 (GK1.5) for 20 min at 4°C. Antibody-bound cells were removed by anti-rat IgG Ab-conjugated magnetic beads (Dynabeads; Dynal). T cell subsets were purified by flow-cytometric sorting (FACS Aria flow cytometer; BD Biosciences).

### Topical association of mice with *S. epidermidis* and lymphocyte isolation from ear skin

*S. epidermidis* strain (NIHLM087) association and skin digests were performed as previously described (Linehan et al., 2018; Park et al., 2019). Briefly, *S. epidermidis* was cultured for 18 h in tryptic soy broth at 37°C. For topical association of bacteria, each mouse was associated by placing 2.5 ml of bacterial suspension (∼2.5 × 10^9^ total bacteria) across the flank and ear skin surface using a sterile cotton swab. Application of bacterial suspension was repeated every second day for 7 d. On day 7, ears were harvested, and skin was incubated in Liberase TL/DNase solution (0.25 mg/ml Liberase; Sigma-Aldrich; and 0.1 µg/ml DNase; Sigma-Aldrich) at 37°C for 20 min and the epidermis separated from the dermis, then chopped and incubated for a further 60 min before passing through a 70-µm filter before staining for flow cytometry.

### Oral antibiotic treatment

Mice were provided autoclaved drinking water supplemented with ampicillin (0.5 mg/ml; Sigma-Aldrich), gentamicin (0.5 mg/ml; Sigma-Aldrich), metronidazole (0.5 mg/ml; Sigma-Aldrich), neomycin (0.5 mg/ml; Sigma-Aldrich), vancomycin (0.25 mg/ml; Sigma-Aldrich), and sucralose (4 mg/ml; Splenda; McNeil Nutritionals, LLC; Abt et al., 2012); or ampicillin (1 mg/ml), streptomycin (5 mg/ml), colistin (1 mg/ml; Sigma-Aldrich) and vancomycin (0.25 mg/ml) added to the sterile drinking water of mice (Routy et al., 2018). Splenda was added to make the antibiotic cocktail more palatable. Mice were placed on antibiotic treatment for up to 1 wk before birth and continued for the duration of the experiment. Mice were analyzed at 4 wk of age. Antibiotic activity was confirmed by collecting feces at weekly intervals and bacterial colonization quantified using 16s ribosomal RNA gene copy number determined by real-time PCR (Denman and McSweeney, 2006).

### Ex vivo single-cell multiplex RT-PCR for paired CDR3-β and CDR3-α analysis

Single effector (TCRβ^+^CD8^+^CD44^+^CD62L^−^RORγt^−^) cells from *Tcf7^+/+^Rorc(γt)*^+^GFP mice, MAIT cells (TCRβ^+^CD8^+^CD44^+^CD62L^−^RORγt^+^) from *Tcf7^+/+^Rorc(γt)^+^*GFP mice, and Tc17 (TCRβ^+^CD8^+^CD44^+^CD62L^−^CD25^+^) cells from *Tcf7^−/−^* mice were isolated by sorting single cells with a FACS Aria into 80 wells of a 96-well twin-tec plate (Eppendorf). The CDR3α and CDR3 β regions were determined by a single-cell multiplex RT-PCR (Dash et al., 2011). mRNA transcripts were reverse-transcribed to cDNA using a VILO kit (Invitrogen) according to the manufacturer’s instructions. The CDR3α and CDR3β regions were amplified by nested PCR using 23 TRAV (T cell receptor alpha variable gene) and 19 TRBV (T cell receptor beta variable gene) primers for external and internal rounds of PCR, together with constant region TRAC and T cell receptor beta constant primers (Dash et al., 2011). For the internal round of PCR, 2.5 µl of the external product was used as template, with either a set of TRAV or TRBV internal primers. Positive PCR products were purified with Exo-SAP-IT at 37°C for 15 min and at 80°C for 15 min. Cleaned PCR products were sequenced using TRAC or T cell receptor beta constant internal primers. Sequences were analyzed using FinchTV, and V and J region usage identified using the ImMunoGeneTics information system (http://www.imgt.org/IMGT_vquest). Where dual TCRα or TCRβ transcripts were detected for a single cell, sequencing with the relevant TRAV or TRBV internal primers was performed to separately identify each transcript.

### Chromatin immunoprecipitation

Primers for ChIP-PCR were designed around peaks identified in published TCF-1 ChIP-seq data on thymocytes and published primer sequences for control gene Axin 2 (Zhou et al., 2010). Primer sequences are outlined in Table S2. ChIP analyses were performed on WT thymocytes that were cross-linked with 1% paraformaldehyde and lysed in lysis buffer (1% SDS, 1 mM EDTA, and protease inhibitors). Cross-linked DNA was sonicated with a Bioruptor (Diagenode). Protein G Dynabeads (Invitrogen) were incubated with 20 µg anti–TCF-1 (H-11; Santa Cruz Biotechnology) or 20 µg of purified rabbit IgG. Coupled antibody was added to 200 µg chromatin, followed by incubation at 4°C overnight. Unbound chromatin was removed using a series of four washes. After elution, bound chromatin was reverse–cross-linked and subjected to phenol-chloroform immunoprecipitation. Enrichment of recovered DNA was measured by real-time PCR.

### RNA sequencing and analysis

#### Murine CD8^+^ T cells

CD8^+^ T cell subsets were purified by flow-cytometric sorting (FACS Aria flow cytometer; BD Biosciences) from *Tcf7^−/−^* IL17^+^CD8^+^ Tc17 cells (TCRβ^+^, CD8^+^, CD44^+^, CD62L^−^, and CD25^+^) and naive (TCRβ^+^, CD8^+^, CD44^+^, CD62L^+^, and CD25^−^), effector (TCRβ^+^, CD8^+^, CD44^+^, CD62L^−^, and CD25^−^), and memory (TCRβ^+^, CD8^+^, CD44^+^, CD62L^+^, and CD25^−^) CD8^+^ T cells from *Tcf7^+/+^* and *Tcf7^−/−^* mice. Total RNA was prepared from each subset using an RNeasy mini kit (Qiagen). 2 × 10^5^ T cells and two biological replicates were generated and subjected to 100-bp single-end sequencing on an Illumina HiSeq2000 at the Australian Genome Research Facility (Melbourne, Australia). Around 20 million reads were generated for each replicate and aligned to the GRCm38/mm10 build of the *Mus musculus* genome using the Subread aligner (Liao et al., 2013). Genewise counts were obtained using featureCounts (Liao et al., 2014). Reads overlapping exons in annotation build 38.1 of the National Center for Biotechnology Information (NCBI) RefSeq database were included. Genes were filtered from downstream analysis if they failed to achieve a counts per million (CPM) mapped reads value of ≥0.5 in at least two libraries. Counts were converted to log2-CPM, quantile-normalized, and precision-weighted with the voom function of the limma package (Law et al., 2014; Ritchie et al., 2015). The log2-CPM values were then converted to log2–reads per kilo exonic bases per million mapped reads values. A linear model was fitted to each gene, and empirical Bayes’ moderated *t*-statistics were used to assess differences in expression (Smyth, 2004). Genes were called DE if they achieved a false discovery rate (FDR of ≤0.05 and also had ≥8 reads per kilo exonic bases per million mapped reads in one or both of the two cell types being compared. Functional gene annotation was performed using Metascape analyses (Tripathi et al., 2015).

#### Human T cells

Total RNA was prepared from three biological replicates each using 2 × 10^4^ purified human splenic IL-17^+^CD8^+^ T cells (CD3^+^, CD4^−^, CD8^+^, Vα7.2^−^, TCRδ^−^, and IL-17^+^), IL-17^−^CD8^+^ effector T cells (CD3^+^, CD4^−^, CD8^+^, Vα7.2^−^, TCRδ^−^, and IL-17^−^), IL-17^+^CD4^+^ (Th17: CD3^+^, CD4^+^, CD8^−^, Vα7.2^−^, TCRδ^−^, and IL-17^+^), and IL-17^−^CD4^+^ (T helper type 1: CD3^+^, CD4^+^, CD8^−^, Vα7.2^−^, TCRδ^−^, and IL-17^−^) using an RNeasy micro kit (Qiagen). Libraries were prepared using the SMART-Seq v4 Ultra Low Input RNA kit (Clontech Laboratories). Samples were sequenced on an Illumina NextSeq 500 generating 75 bp paired-end reads. Human RNA-seq data were analyzed in a similar way as the analysis of mouse RNA-seq data. Genes were called DE if they achieved an FDR of ≤0.01.

### ATAC sequencing and analysis

CD8^+^ T cell subsets were purified by flow-cytometric sorting (FACS Aria flow cytometer, BD Biosciences) from naive WT and *Tcf7^−/−^* mice. For each biological replicate (*n* = 2), 5 × 10^4^ cells of each subset were prepared for sequencing as previously described (Buenrostro et al., 2013, 2015). Samples were sequenced on an Illumina NextSeq 500 generating 75 bp paired-end reads. Reads were aligned to the GRCm38/mm10 build of the *M. musculus* genome using the Subread aligner. Only uniquely mapped reads were retained. ATAC-seq peaks were called using Macs2 with an FDR cutoff of 0.01. Overlapping peaks called from different samples were merged into a single peak region. Mapped reads in each sample were then assigned to these regions using featureCounts. Region counts were converted to log2-CPM and quantile-normalized with the voom method in limma. Regions were assigned to genes based on the overlap between genomic span of a region and gene body that is extended 20 kb upstream and 5 kb downstream. To determine enriched transcription factor binding motifs, de novo motif analysis using Homer was applied to accessible chromatin regions unique to Tc17 cells.

### Analysis of chromatin immunoprecipitation sequencing

ChIP-seq data for TCF-1 has been published previously (Dose et al., 2014) and was downloaded from the GEO database (accession no. GSE46662). Sequence reads were aligned to the GRCm38/mm10 build of the *M. musculus* genome using the Subread aligner, and only uniquely mapped reads were retained. Binding peaks were called using the Homer program (Heinz et al., 2010). Chromatin input control was used to test the significance of binding enrichment. An FDR cutoff 1 ×10^−5^ was applied. Called peaks were assigned to a gene if they overlapped with the body of the gene plus 20 kb upstream and 5 kb downstream regions. The NCBI RefSeq mouse gene annotation (build 38.1) was used in the assignment of peaks to genes.

### Visualization using tSNE plots

Unbiased clustering tSNE (van der Maaten and Hinton, 2008) were generated using FlowJo software to map multiparameter expression of CD4, CD8, CD62L, CD44, and RORγt in splenic TCRβ^+^ cells from the spleen of WT (*Tcf7^+/+^*) and *Tcf7^−/−^* mice and analyzed by flow cytometry. Data are displayed as a pseudo dot plot where color intensity indicates increasing cell frequency.

### Classification of human tumor samples from TCGA

To obtain classification signatures for Tc1, Tc17, T helper type 1, and Th17 cell line models, two gene expression–based classifiers were developed using Random Forest (Liaw and Weiner, 2002) and Nearest Shrunken Centroid (Tibshirani et al., 2002) algorithms. While the Random Forest classifier is capable of capturing nonlinear relationships between the genes that characterize different subpopulations of T cells, the shrunken centroid classifier captures linear associations. The shrunken centroid classifier implemented in the pamr CRAN package (Hastie et al., 2014) was trained on human splenic T cell RNA-seq data using a threefold cross-validation training procedure with the default parameters. The Random Forest classifier implemented in the randomForest CRAN package was trained using 500 trees, with three genes chosen at random at each split. The default parameters were used for the remaining parameters of the algorithm. Each classifier was then applied to a total number of 593 cancer samples in the TCGA Colon and Rectal Cancer datasets to predict if a sample in the Colorectal Cancer cohort is likely to have an expression profile resembling any of the four T cell sub-types. The samples predicted independently by both classifiers to resemble Tc17 expression profiles were considered to be the ones most likely to contain Tc17 infiltrating cells. To validate the likely Tc17 infiltration in these TCGA samples, we next tested whether these samples are enriched for genes DE in Tc17 splenic cells in our RNA-seq data. We separated putative Tc17 infiltrated samples from other tumor samples, discarding any samples that had a Tc17 prediction by only a single classifier, and performed a differential expression analysis between these human tumor sets using the voom function in limma (Law et al., 2014; Ritchie et al., 2015). The enrichment of the splenic Tc17 gene expression in TCGA gene expression data was then assessed using the mroast gene set enrichment test in limma using genes DE between splenic Tc17 cells and combined other T cell types as the gene set.

### Quantification and statistical analyses

Statistical parameters including the exact value of *n*, the definition of center, dispersion, and precision measures (mean ± SEM), and statistical significance are reported in the figures and figure legends. Analyses were performed using GraphPad Prism software (version 7.0). Data were considered statistically significant when P < 0.05 by Student’s *t* test, Mann Whitney *U* test, or ANOVA with Kruskal–Wallis test.

### Data and materials availability

Raw data files for the RNA sequencing and ATAC sequencing have been deposited in the NCBI Gene Expression Omnibus under accession nos. GSE83841, GSE96741, and GSE119839.

### Online supplemental material

Fig. S1 shows the transcriptional and functional phenotype of Tc17 cells at steady state and following treatment with antibiotics. Fig. S2 shows the gene network analysis of *Tcf7^−/−^* Tc17 cells. Fig. S3 shows TCF-1 changes in chromatin accessibility of genes required for function of effector CD8^+^ T cells. Fig. S4 shows the deletion efficiency and T cell development in *Tcf7^fl/fl^CD4Cre^+/T^* mice. Fig. S5 shows the differential gene expression in human IL-17^−^CD4^+^ versus IL-17^+^CD4^+^ cells. Table S1 shows de novo motif enrichment using Homer from ATAC-seq peaks enriched in Tc17 cells, and Table S2 shows primer sequences for quantitative ChIP-PCR analysis.

## Supplementary Material

Supplemental Materials (PDF)
