# Supplementary material for: TCF-1 limits the formation of Tc17 cells via repression of the MAF–RORγt axis
Source: J Exp Med. 2019 May 29;216(7):1682–99. doi: 10.1084/jem.20181778 (PMC6605755; doi:10.1084/jem.20181778)
Supplement: Supplemental Materials (PDF) [file JEM_20181778_sm.pdf]

Supplemental material

Mielke et al., https://doi.org/10.1084/jem.20181778

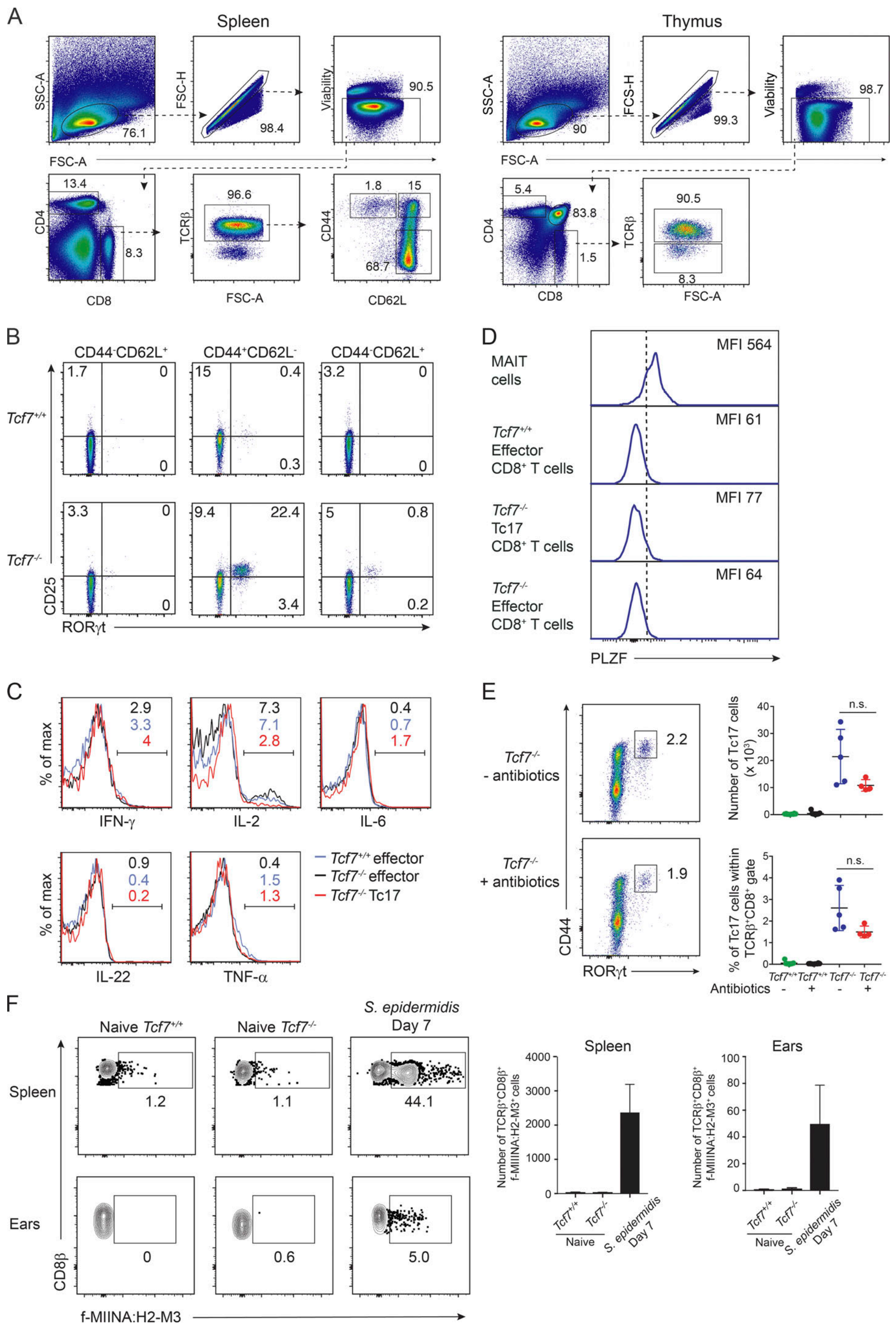

Figure S1. **Transcriptional and functional phenotype of Tc17 cells. (A)** Representative gating strategy for CD4⁺ and CD8⁺ T cell from mouse spleen and thymus. **(B)** RORγt and CD25 expression in WT and *Tcf7*$^{-/-}$ naive (TCRβ$^+$CD4$^-$CD8$^+$CD44$^-$CD62L$^+$), effector (TCRβ$^+$CD4$^-$CD8$^+$CD44$^+$CD62L$^-$), and memory (TCRβ$^+$CD4$^-$CD8$^+$CD44$^+$CD62L$^+$) CD8$^+$ T cells. Data show one of three similar experiments. **(C)** Intracellular staining for cytokine expression in WT and *Tcf7*-deficient effector cells (TCRβ$^+$CD4$^-$CD8$^+$CD44$^+$CD62L$^-$RORγt$^-$) and Tc17 cells (TCRβ$^+$CD4$^-$CD8$^+$CD44$^+$CD62L$^-$RORγt$^+$) stimulated for 4 h with PMA and ionomycin in the presence of Brefeldin A. Data show one of two experiments with similar results (*n* = 4 mice/genotype). The frequency of cells expressing each cytokine is shown. **(D)** PLZF expression in *Tcf7*$^{+/+}$ MAIT cells (TCRβ$^+$CD4$^-$CD8$^+$CD44$^+$CD62L$^-$RORγt$^+$), *Tcf7*$^{+/+}$ and *Tcf7*$^{-/-}$ effector cells (TCRβ$^+$CD4$^-$CD8$^+$CD44$^+$CD62L$^-$RORγt$^-$), and *Tcf7*$^{-/-}$ Tc17 cells (TCRβ$^+$CD4$^-$CD8$^+$CD44$^+$CD62L$^-$CD25$^+$RORγt$^+$) isolated from spleen. Data show representative profiles of one of two experiments with similar results (*n* = 4 mice/genotype). **(E)** Pregnant *Tcf7*$^{+/+}$ or *Tcf7*$^{-/-}$ mice were treated with a combination of antibiotics in their drinking water, or left untreated, for up to 1 wk before, and continued for 4 wk after birth. Flow-cytometric analyses (left panels) show dot plots of Tc17 cells from antibiotic treated and untreated mice. Data are representative of two similar experiments (*n* = 4–9 mice/group for each experiment). Total number and frequency of Tc17 cells in spleen of 4-wk-old pups (right panels) showing the mean ± SD (*n* = 4–9 mice/group for each experiment). P values were calculated using ANOVA with Kruskal–Wallis test. **(F)** Representative contour plots (left panels) and total cell number (right panels) of f-MIINA:H2-M3 tetramer$^+$ CD8$^+$ T cells from spleen and ears of naive *Tcf7*$^{+/+}$, *Tcf7*$^{-/-}$ and WT mice 7 d after association with *S. epidermidis*. Contour plots are gated on live TCRβ$^+$CD8β$^+$ cells. Histograms show the mean ± SD of samples pooled from one of two similar experiments (*n* = 2–4 mice per group per experiment).SSC-A, side scatter-area; FSC-H, forward scatter height; FSC-A, forward scatter area.

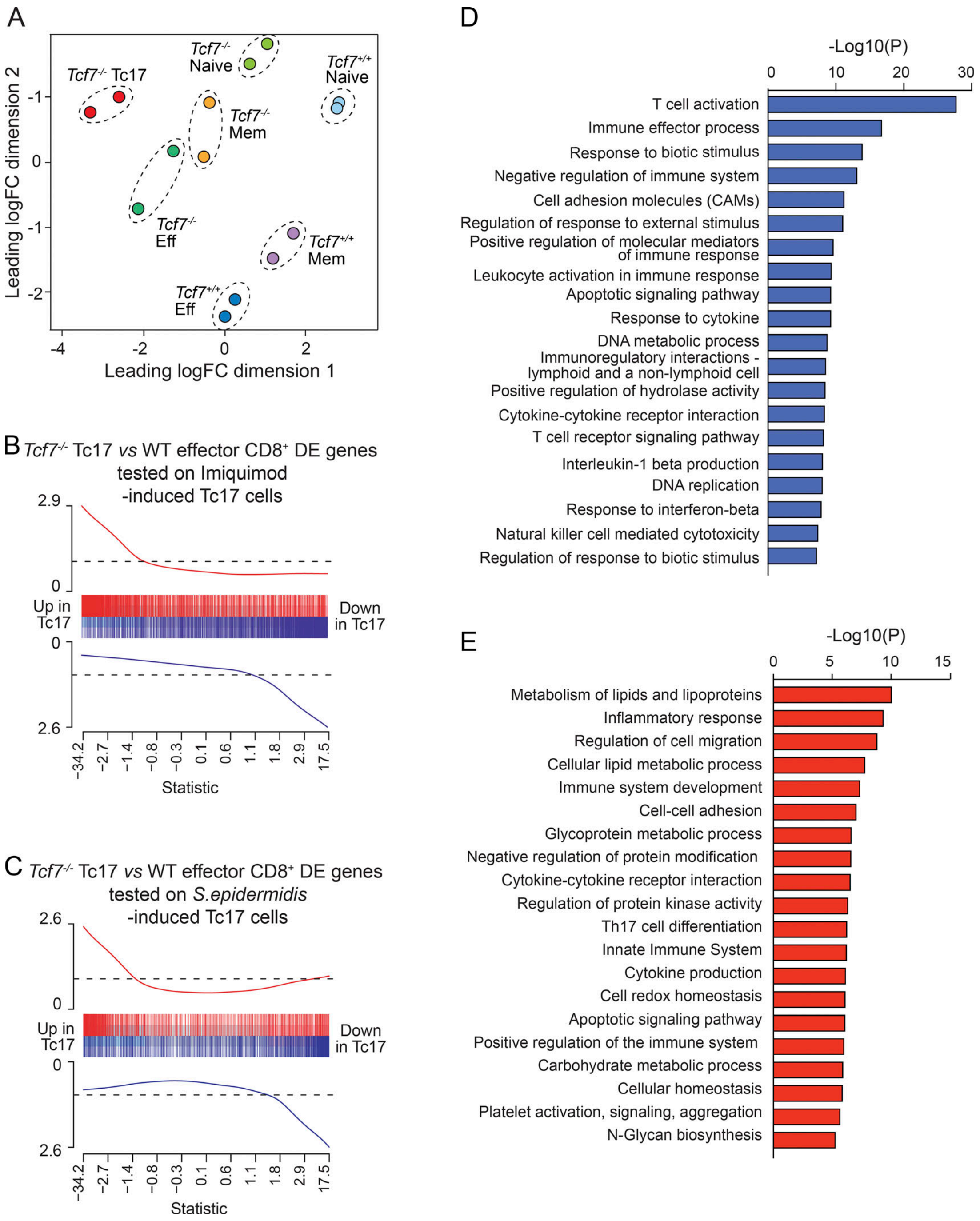

Figure S2. **Gene network analysis of *Tcf7*$^{-/-}$ Tc17 cells. (A)** Multidimensional scaling analysis of RNA-seq data from *Tcf7*$^{-/-}$ Tc17 cells (TCRβ$^+$CD4$^-$CD8$^+$CD44$^+$CD62L$^-$CD25$^+$) and naive (TCRβ$^+$CD4$^-$CD8$^+$CD44$^+$CD62L$^-$CD25$^-$), effector (TCRβ$^+$CD4$^-$CD8$^+$CD44$^+$CD62L$^-$CD25$^-$), and memory (TCRβ$^+$CD4$^-$CD8$^+$CD44$^+$CD62L$^+$CD25$^-$) CD8$^+$ T cells from *Tcf7*$^{+/+}$ and *Tcf7*$^{-/-}$ mice. Each point represents an individual sample and samples are color coded by population. Distances on the plot show the leading log2-fold change (logFC) between samples. **(B and C)** Gene set enrichment analysis comparing *Tcf7*$^{-/-}$ Tc17 versus WT effector cell DE genes with DE genes in B, imiquimod-induced skin Tc17 cells versus effector CD8$^+$ T cells (left panel, $P < 0.0001$) and C, DE genes in topical *S. epidermidis*–induced Tc17 versus effector CD8$^+$ T cells (Linehan et al., 2018; right panel, $P < 0.0001$). **(D and E)** Top 20 gene ontology categories defined by Metascape analysis ranked by P value (log10) for DE genes that were down-regulated (D) and up-regulated (E) in *Tcf7*$^{-/-}$ Tc17 cells.

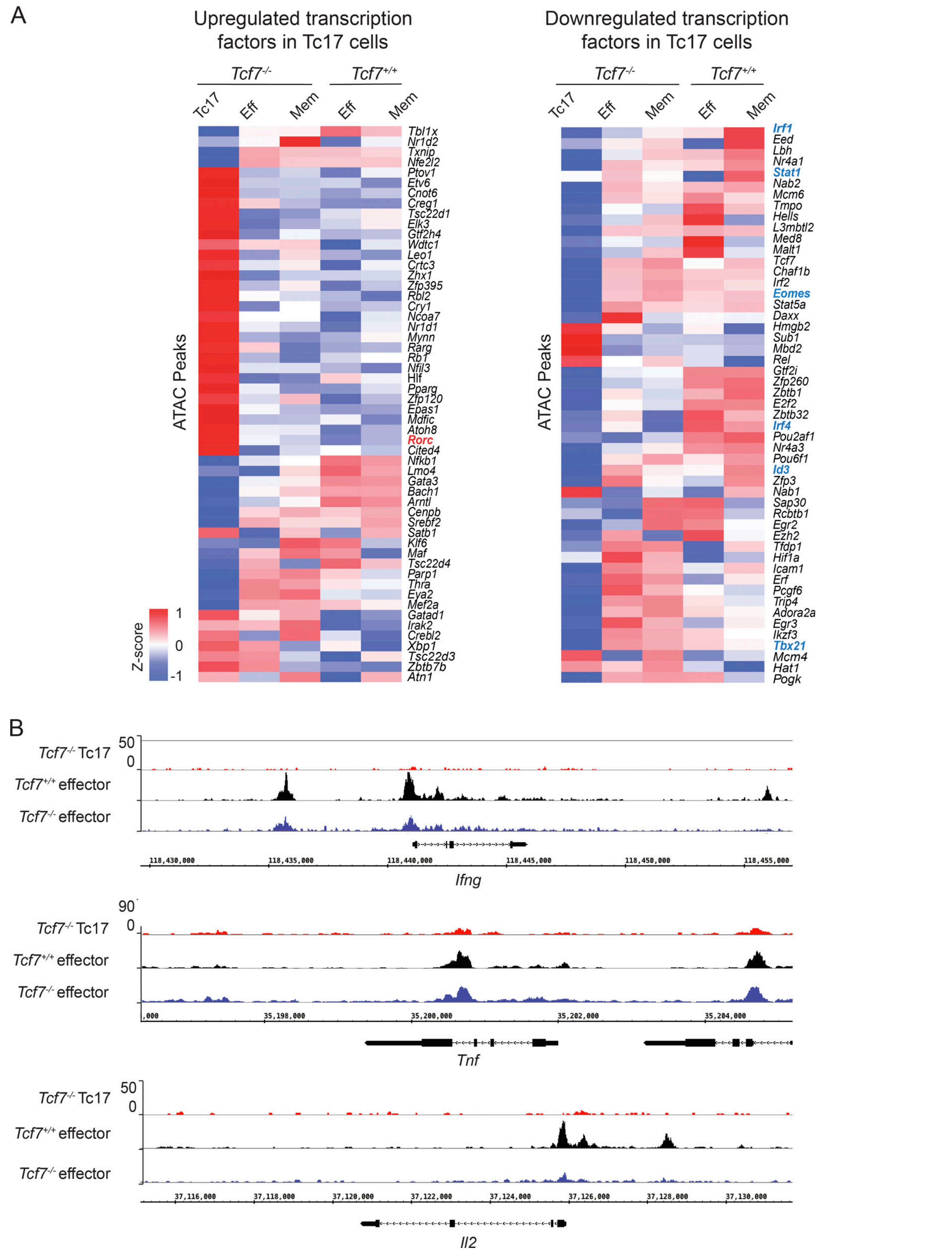

Figure S3. **TCF-1 enhances chromatin accessibility of genes required for function of effector CD8⁺ T cells. (A)** Heat map of regions of chromatin accessibility across the genomes of *Tcf7*$^{-/-}$ Tc17 cells (TCRβ$^+$CD4$^-$CD8$^+$CD44$^+$CD62L$^-$CD25$^+$), effector (TCRβ$^+$CD4$^-$CD8$^+$CD44$^+$CD62L$^-$CD25$^-$), and memory (TCRβ$^+$CD4$^-$CD8$^+$CD44$^+$CD62L$^+$CD25$^-$) CD8$^+$ T cells from *Tcf7*$^{+/+}$ and *Tcf7*$^{-/-}$ mice. Average relative peak abundance (Z-score) calculated from two independent biological replicates from log2-CPM value of peak regions is shown, color-coded according to the legend. Rows are scaled to have a mean of 0 and an SD of 1. Selected transcription factors are shown that display an increased gene expression in Tc17 cells (left panel) or decreased gene expression in Tc17 cells compared with WT effector CD8$^+$ T cells (right panel). **(B)** Genome browser of normalized ATAC-seq reads across the *Ifng, Tnf, and Il2 loci* from *Tcf7*$^{-/-}$ Tc17 cells, and effector cells from *Tcf7*$^{+/+}$ and *Tcf7*$^{-/-}$ mice.

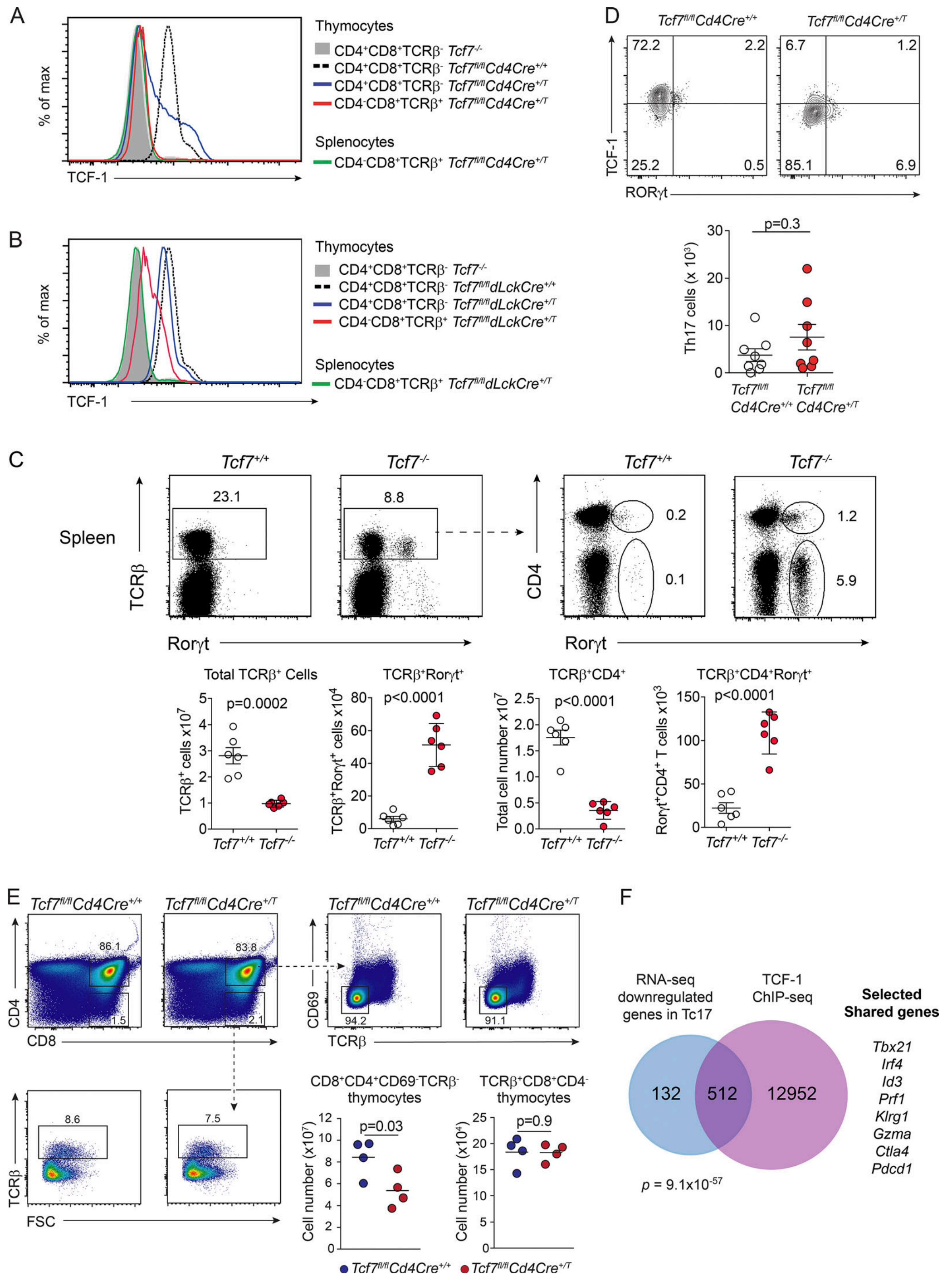

Figure S4. **Deletion efficiency and T cell development in $Tcf7^{fl/fl}CD4Cre^{+/T}$ mice. (A and B)** Flow-cytometric profiles of expression of TCF-1 in thymocytes and splenocytes from (A) $Tcf7^{fl/fl}CD4Cre^{+/T}$ and (B) $Tcf7^{fl/fl}dLckCre^{+/T}$ mice. Data show representative profiles from one of two independent experiments (n = 4/genotype). **(C)** Flow-cytometric marker expression, including RORγt in $CD4^+$ T cells isolated from the spleen of WT and $Tcf7^{-/-}$ mice (top panel). Total cell numbers are shown in the bottom panel. Data show representative plots (upper panels) and the mean ± SEM of individuals pooled from three independent experiments (lower panels; n = 6; P values calculated using Student's t test). **(D)** Flow-cytometric analyses of Th17 ($TCR\beta^+CD4^+CD8^-Ror\gamma t^+$) cells isolated from the spleen of $Tcf7^{fl/fl}CD4Cre^{+/+}$ and $Tcf7^{fl/fl}CD4Cre^{+/T}$ mice (upper panels). Enumeration of Th17 cells (lower panel) showing the mean ± SEM of samples pooled from two independent experiments (n = 8 mice/genotype). **(E)** Representative dot plots and total number of DP thymocytes ($CD8^+CD4^+CD69^-TCR\beta^-$) and mature $CD8^+$ T cells ($TCR\beta^+CD8^+CD4^-$) from $Tcf7^{fl/fl}CD4Cre^{+/T}$ and control mice. Data are representative of two independent experiments (n = 4 mice/group/experiment). Exact P values were calculated using the Mann–Whitney U test. **(F)** Venn diagram analyses of down-regulated genes in Tc17 cells ($Tcf7^{-/-}$ Tc17 cells versus $Tcf7^{+/+}$ effector $CD8^+$ T cells from RNA-seq analysis compared with TCF-1 target genes identified by published ChIP-seq datasets; Dose et al., 2014). Selected shared TCF-1 target genes down-regulated by Tc17 cells are shown.

https://doi.org/10.1084/jem.20181778

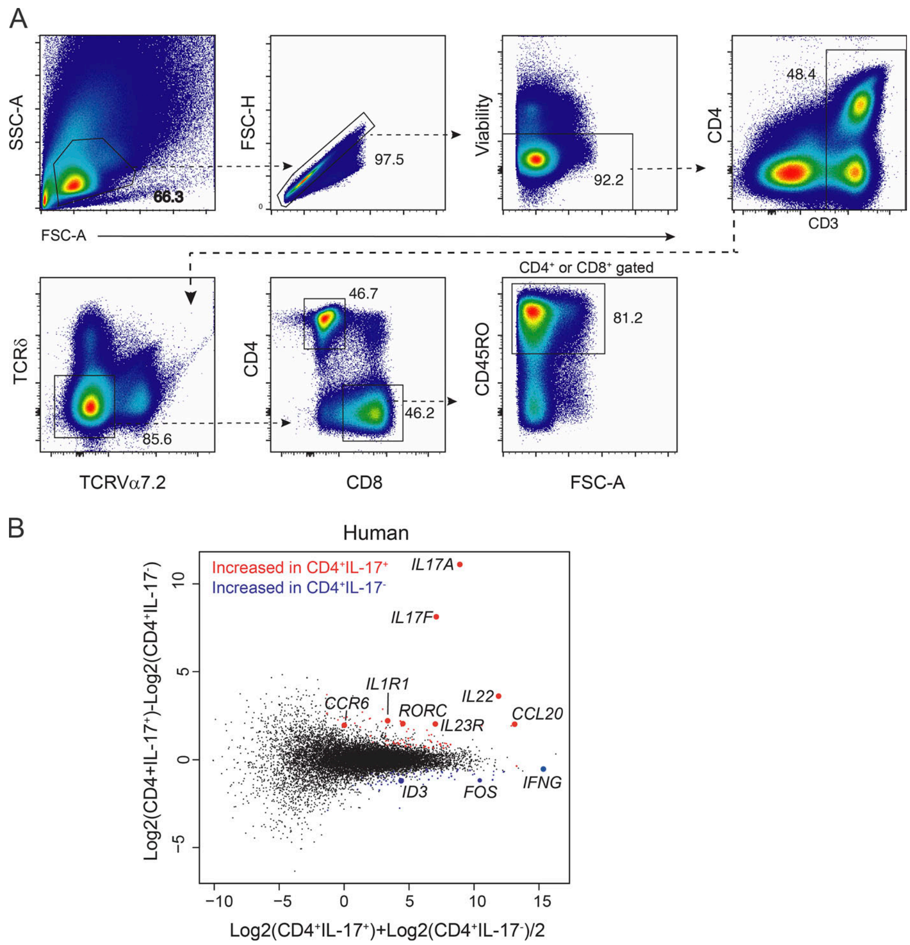

Figure S5. **Differential gene expression in human IL-17⁻ CD4⁺ versus IL-17⁺ CD4⁺ cells. (A)** Representative gating strategy of human spleen CD8⁺ and CD4⁺ T cells. **(B)** Mean difference plot highlighting genes significantly increased in human spleen IL-17⁺CD4⁺ cells (red, CD3⁺CD4⁺CD8⁻Vα7.2⁻TCRγδ⁻IL-17⁺) versus genes increased in human spleen IL-17⁻ CD4⁺ cells (blue, CD3⁺CD4⁺CD8⁻Vα7.2⁻TCRγδ⁻IL-17⁻). Data show log2 fold change compared with mean expression.

Table S1. **De novo motif enrichment using HOMER from ATAC-seq peaks enriched in Tc17 cells**

Motif name	Consensus (5′- and -3′)	P value	% of target sequences with motif
Fli1 (ETS)	NRYTTCCGGH	1e-557	51.72%
ETS1 (ETS)	ACAGGAAGTG	1e-511	49.23%
ERG (ETS)	ACAGGAAGTG	1e-442	59.59%
RORgt (NR)	AAYTAGGTCA	1e-438	13.96%
Ets1-distal (ETS)	MACAGGAAGT	1e-437	23.11%
GABPA (ETS)	RACCGGAAGT	1e-434	43.29%
ETV1 (ETS)	AACCGGAAGT	1e-427	55.53%
EWS:FLI1-fusion (ETS)	VACAGGAAAT	1e-417	32.03%
EWS:ERG-fusion (ETS)	ATTTCCTGTN	1e-382	34.31%
CTCF (Zf)	AYAGTGCCMYCTRGTGGCCA	1e-328	14.17%
RUNX-AML (Runt)	GCTGTGGTTW	1e-315	33.49%
RUNX1 (Runt)	AAACCACARM	1e-312	42.86%
RUNX (Runt)	SAAACCACAG	1.00E-307	33.69%
Elk1 (ETS)	HACTTCCGGY	1.00E-299	29.15%
ETS (ETS)	AACCGGAAGT	1.00E-270	20.64%
ELF1 (ETS)	AVCCGGAAGT	1.00E-267	26.90%
Elk4 (ETS)	NRYTTCCGGY	1.00E-262	27.73%
RUNX2 (Runt)	NWAACCACADNN	1.00E-260	36.45%
ETS:RUNX (ETS,Runt)	RCAGGATGTGGT	1.00E-253	9.82%
BORIS (Zf)	CNNBRGCGCCCCCTGSTGGC	1.00E-237	15.75%
EHF (ETS)	AVCAGGAAGT	1.00E-229	45.74%
PU.1 (ETS)	AGAGGAAGTG	1.00E-227	24.81%
SPDEF (ETS)	ASWTCCTGBT	1.00E-184	37.61%
ELF5 (ETS)	ACVAGGAAGT	1.00E-148	28.70%
Sp1 (Zf)	GGCCCCGCCCCC	1.00E-59	11.65%
SpiB (ETS)	AAAGRGGAAGTG	1.00E-53	9.28%
GATA (Zf)	NAGATWNBNATCTNN	1.00E-46	3.66%
Gata1 (Zf)	SAGATAAGRV	1.00E-43	16.29%
Gata2 (Zf)	BBCTTATCTS	1.00E-43	17.86%
Gata4 (Zf)	NBWGATAAGR	1.00E-39	24.99%
GATA3 (Zf)	AGATAASR	1.00E-38	34.52%
GATA (Zf),IR3	NNNNNBAGATAWYATCTVHN	1.00E-33	4.93%
Atf1 (bZIP)	GATGACGTCA	1.00E-32	16.90%
IRF2 (IRF)	GAAASYGAAASY	1.00E-30	4.03%
IRF1 (IRF)	GAAAGTGAAAGT	1.00E-28	4.85%
PU.1-IRF (ETS:IRF)	MGGAAGTGAAAC	1.00E-28	33.83%
RFX (HTH)	CGGTTGCCATGGCAAC	1.00E-27	3.70%
Rfx2 (HTH)	GTTGCCATGGCAACM	1.00E-26	3.92%
Klf4 (Zf)	GCCACACCCA	1.00E-25	13.24%
KLF5 (Zf)	DGGGYGKGGC	1.00E-25	34.64%
Reverb (NR),DR2	GTRGGTCASTGGGTCA	1.00E-25	5.01%
Atf7 (bZIP)	NGRTGACGTCAY	1.00E-25	12.18%
Atf2 (bZIP)	NRRTGACGTCAT	1.00E-23	8.98%
Jun-AP1 (bZIP)	GATGASTCATCN	1.00E-23	6.72%

Table S1. **De novo motif enrichment using HOMER from ATAC-seq peaks enriched in Tc17 cells (*Continued*)**

Motif name	Consensus (5′- and -3′)	P value	% of target sequences with motif
Atf3 (bZIP)	DATGASTCATHN	1.00E-22	15.45%
E2F4 (E2F)	GGCGGGAAAH	1.00E-21	11.35%
Foxo1 (Forkhead)	CTGTTTAC	1.00E-21	42.69%
Fra1 (bZIP)	NNATGASTCATH	1.00E-20	12.97%
Bach2 (bZIP)	TGCTGAGTCA	1.00E-20	5.75%
Fosl2 (bZIP)	NATGASTCABNN	1.00E-19	8.48%
GFY (?)	ACTACAATTCCC	1.00E-19	2.76%
ISRE (IRF)	AGTTTCASTTTC	1.00E-19	2.38%
JunD (bZIP)	ATGACGTCATCN	1.00E-19	3.16%
CRE (bZIP)	CSGTGACGTCAC	1.00E-18	6.55%
c-Jun-CRE (bZIP)	ATGACGTCATCY	1.00E-17	7.85%
ELT-3 (Gata)	AWTGATAAGA	1.00E-17	10.31%
X-box (HTH)	GGTTGCCATGGCAA	1.00E-17	3.82%
AP-1 (bZIP)	VTGACTCATC	1.00E-16	16.98%
STAT1 (Stat)	NATTTCCNGGAAAT	1.00E-16	8.03%
Usf2 (bHLH)	GTCACGTGGT	1.00E-16	8.93%
PQM-1 (?)	ACTGATAAGA	1.00E-16	8.96%
EKLF (Zf)	NWGGGTGTGGCY	1.00E-15	7.16%
IRF4 (IRF)	ACTGAAACCA	1.00E-15	11.32%
Bach1 (bZIP)	AWWNTGCTGAGTCAT	1.00E-15	2.02%
Nrf2 (bZIP)	HTGCTGAGTCAT	1.00E-15	1.88%
MafA (bZIP)	TGCTGACTCA	1.00E-15	21.87%
MITF (bHLH)	RTCATGTGAC	1.00E-14	22.45%
NF-E2 (bZIP)	GATGACTCAGCA	1.00E-14	2.13%
Rfx1 (HTH)	KGTTGCCATGGCAA	1.00E-14	6.21%
CTCF-SatelliteElement (Zf?)	TGCAGTTCCMVNWRTGGCCA	1.00E-13	1.00%
Unknown-ESC-element (?)	CACAGCAGGGGG	1.00E-13	15.40%
STAT5 (Stat)	RTTTCTNAGAAA	1.00E-12	8.91%
E-box (bHLH)	SSGGTCACGTGA	1.00E-12	2.69%
USF1 (bHLH)	SGTCACGTGR	1.00E-11	11.71%
BATF (bZIP)	DATGASTCAT	1.00E-11	14.67%
Cbf1 (bHLH)	TCACGTGAYH	1.00E-11	7.11%
ETS:E-box (ETS,bHLH)	AGGAARCAGCTG	1.00E-11	2.76%
E2F6 (E2F)	GGCGGGAARN	1.00E-11	12.15%
GATA3 (Zf),DR4	AGATGKDGAGATAAG	1.00E-10	2.52%
SeqBias: A/T bias	WWWWWWWWWW	1.00E-09	94.65%
c-Myc (bHLH)	VCCACGTG	1.00E-09	11.93%
Stat3+il21 (Stat)	SVYTTCCNGGAARB	1.00E-09	17.54%
NRF1 (NRF)	CTGCGCATGCGC	1.00E-09	4.96%
DPL-1 (E2F)	TAGCGCGC	1.00E-09	15.89%
E-box	GCCACGTG	1.00E-09	10.78%
E2F1 (E2F)	CWGGCGGGAA	1.00E-09	5.67%
GFY-Staf (?,Zf)	RACTACAATTCCCAGAAKGC	1.00E-08	2.38%
FOXP1 (Forkhead)	NYYTGTTTACHN	1.00E-08	10.33%

Table S1. **De novo motif enrichment using HOMER from ATAC-seq peaks enriched in Tc17 cells (*Continued*)**

Motif name	Consensus (5′- and -3′)	P value	% of target sequences with motif
bHLHE40 (bHLH)	KCACGTGMCN	1.00E-08	8.01%
STAT4 (Stat)	NYTTCCWGGAAR	1.00E-08	21.37%
Stat3 (Stat)	CTTCCGGGAA	1.00E-08	12.61%
Egr2 (Zf)	NGCGTGGGCGGR	1.00E-07	5.54%
Rfx5 (HTH)	SCCTAGCAACAG	1.00E-07	10.33%
MafK (bZIP)	GCTGASTCAGCA	1.00E-07	7.00%
Unknown5/Drosophila-Promoters/	GCTGATAASV	1.00E-07	13.67%
CLOCK (bHLH)	GHCACGTG	1.00E-07	13.60%
E2F7 (E2F)	VDTTTCCCGCCA	1.00E-06	3.05%
GFX (?)	ATTCTCGCGAGA	1.00E-06	0.48%
Unknown3	AYTAAACCGG	1.00E-06	3.78%
Atf4 (bZIP)	MTGATGCAAT	1.00E-06	5.40%
NFkB-p50,p52 (RHD)	GGGGGAATCCCC	1.00E-06	2.81%
NF1 (CTF)	CYTGGCABNSTGCCAR	1.00E-05	8.78%
GATA:SCL (Zf,bHLH)	CRGCTGBNGNSNNSAGATAA	1.00E-05	3.12%
MafF (bZIP)	HWWGTCAGCAWWTTT	1.00E-05	5.86%
FHY3 (FAR1)	HHCACGCGCBTN	1.00E-05	6.97%
Tbx20 (T-box)	GGTGYTGACAGS	1.00E-05	5.80%
NFAT:AP1 (RHD,bZIP)	SARTGGAAAAWRTGAGTCAB	1.00E-04	3.70%
BMYB (HTH)	NHAACBGYYV	1.00E-04	32.68%
ABF1	CGTRNAAARTGA	1.00E-04	4.34%
ZBTB33 (Zf)	GGVTCTCGCGAGAAC	1.00E-04	1.18%
Chop (bZIP)	ATTGCATCAT	1.00E-04	4.11%
NFkB-p65-Rel (RHD)	GGAAATTCCC	1.00E-04	1.33%
Tcf12 (bHLH)	VCAGCTGYTG	1.00E-04	22.52%
NFkB-p65 (RHD)	WGGGGATTTCCC	1.00E-04	10.51%
CEBP:AP1 (bZIP)	DRTGTTGCAA	1.00E-03	14.56%
STAT6 (Stat)	TTCCKNAGAA	1.00E-03	11.55%
Maz (Zf)	GGGGGGGG	1.00E-03	29.82%
NRF (NRF)	STGCGCATGCGC	1.00E-03	5.57%
VDR (NR)	ARAGGTCANWGAGTTCANNN	1.00E-03	5.10%
AMYB (HTH)	TGGCAGTTGG	1.00E-03	33.52%
CEBP:CEBP (bZIP)	NTNATGCAAYMNNHTGMAAY	1.00E-02	2.81%
REB1	KCCGGGTAAYRR	1.00E-02	3.67%
PRDM9 (Zf)	ADGGYAGYAGCATCT	1.00E-02	9.79%
Foxa2 (Forkhead)	CYTGTTTACWYW	1.00E-02	16.62%
PBX1 (Homeobox)	GSCTGTCACTCA	1.00E-02	2.24%
T1ISRE (IRF)	ACTTTCGTTTCT	1.00E-02	0.42%
GEI-11 (Myb?)	CCGACAYYTYACGGG	1.00E-02	1.19%
Tcf3 (HMG)	ASWTCAAAGG	1.00E-02	6.14%
Tcfcp2l1 (CP2)	NRAACCRGTTYRAACCRGYT	1.00E-02	3.89%
PRDM1 (Zf)	ACTTTCACTTTC	1.00E-02	12.31%
HIF2a (bHLH)	GCACGTACCC	1.00E-02	7.69%
MyoD (bHLH)	RRCAGCTGYTSY	1.00E-02	17.80%

Table S1. **De novo motif enrichment using HOMER from ATAC-seq peaks enriched in Tc17 cells (*Continued*)**

Motif name	Consensus (5′- and -3′)	P value	% of target sequences with motif
Pho4 (bHLH)	AAGCACGTGBGD	1.00E-02	4.71%
Pbx3 (Homeobox)	SCTGTCAMTCAN	1.00E-02	5.75%

Table S2. **Primer sequences for quantitative ChIP-PCR analysis**

Genome locations	5′ primer	3′ primer
Maf-1	GCCTCTGATCCCAGCGAGAA	TGCTAATCGCTGCCGCTCTC
Maf-2	AGAGACCTGTGCCTGTTGAT	GATGTCTCCTCTATCACTGG
Maf-3	TGGAAGCTTCTGAGGGCTTT	ACTCAAGCCCTCTCCAGAAC
Maf-4	CTCAGCTGGGCTCTCTGCTC	TGAGGAGATGGCTATGCCTCC
Maf-5	GGTTGTAGCAGCTACCAAAC	ACTCTTGTAGGTCCTTGGCA
Axin2-1	TGTGTGGAGCTCAGATTTCG	ATGTGAGCCTCCTCTCTGGA
Axin2-2	AAATCCACAGCGCAGTTTTT	TTCAACCCAGGTCCTGTTTC

References

Dose, M., A.O. Emmanuel, J. Chaumeil, J. Zhang, T. Sun, K. Germar, K. Aghajani, E.M. Davis, S. Keerthivasan, A.L. Bredemeyer, et al. 2014. β-Catenin induces T-cell transformation by promoting genomic instability. *Proc. Natl. Acad. Sci. USA.* 111:391–396. https://doi.org/10.1073/pnas.1315752111

Linehan, J.L., O.J. Harrison, S.J. Han, A.L. Byrd, I. Vujkovic-Cvijin, A.V. Villarino, S.K. Sen, J. Shaik, M. Smelkinson, S. Tamoutounour, et al. 2018. Non-classical Immunity Controls Microbiota Impact on Skin Immunity and Tissue Repair. *Cell.* 172:784–796. https://doi.org/10.1016/j.cell.2017.12.033
